# *Salmonella* Typhimurium Type III Secretion Effectors Stimulate Innate Immune Responses in Cultured Epithelial Cells

**DOI:** 10.1371/journal.ppat.1000538

**Published:** 2009-08-07

**Authors:** Vincent M. Bruno, Sebastian Hannemann, María Lara-Tejero, Richard A. Flavell, Steven H. Kleinstein, Jorge E. Galán

**Affiliations:** 1 Section of Microbial Pathogenesis, Yale University School of Medicine, New Haven, Connecticut, United States of America; 2 Department of Immunobiology, Howard Hughes Medical Institute, Yale University School of Medicine, New Haven, Connecticut, United States of America; 3 Department of Pathology and Interdepartmental Program in Computational Biology and Bioinformatics, Yale University School of Medicine, New Haven, Connecticut, United States of America; Tufts University School of Medicine, United States of America

## Abstract

Recognition of conserved bacterial products by innate immune receptors leads to inflammatory responses that control pathogen spread but that can also result in pathology. Intestinal epithelial cells are exposed to bacterial products and therefore must prevent signaling through innate immune receptors to avoid pathology. However, enteric pathogens are able to stimulate intestinal inflammation. We show here that the enteric pathogen *Salmonella* Typhimurium can stimulate innate immune responses in cultured epithelial cells by mechanisms that do not involve receptors of the innate immune system. Instead, *S.* Typhimurium stimulates these responses by delivering through its type III secretion system the bacterial effector proteins SopE, SopE2, and SopB, which in a redundant fashion stimulate Rho-family GTPases leading to the activation of mitogen-activated protein (MAP) kinase and NF-κB signaling. These observations have implications for the understanding of the mechanisms by which *Salmonella* Typhimurium induces intestinal inflammation as well as other intestinal inflammatory pathologies.

## Author Summary

*Salmonella* Typhimurium is one of the most common causes of food-borne illness in the United States and a major cause of diarrheal diseases in developing countries. This pathogen induces diarrhea by stimulating inflammation in the intestinal tract. This study shows that *S.* Typhimurium delivers molecules into epithelial cells with the capacity to stimulate the production of pro-inflammatory substances. This mechanism may help the pathogen to initiate the inflammatory response in the intestinal epithelium. This study provides insight into the mechanisms by which *Salmonella* Typhimurium causes diarrhea.

## Introduction

It is widely believed that one of the main triggers of host inflammation is the recognition of microbial products by receptors of the innate immune system [1]–[3]. Conserved microbial products, collectively known as “pathogen-associated molecular patterns” (PAMPS), stimulate specific host receptors triggering a variety of generic responses directed at controlling pathogen spread. In the case of bacterial pathogens, conserved bacterial products such as lipopolysacharide, flagella, or peptidoglycan, are recognized by transmembrane Toll-like receptors (TLRs), or intracellular nucleotide oligomerizaton domain-like receptors (NLRs), leading to conserved signaling cascades that culminate in the activation of NF-*κ*B, mitogen-activated protein kinases (MAPK), and the production of pro-inflammatory cytokines [4]–[7]. These receptors are widely expressed in cells of the innate immune system such as macrophages, dendritic cells, and neutrophils, which consequently are well equipped to respond to virtually any invading pathogen.

Intestinal epithelial cells, however, are a special case in that they are exposed to massive amounts of bacterial products with the potential to activate innate immune receptors. Therefore, signaling through these receptors, in particular surface TLRs, must be prevented to avoid uncontrolled inflammation, which would be detrimental to the host. The mechanisms by which this negative regulation of innate immune receptor activation is exerted are poorly understood. It is believed that a combination of topological sequestration of the receptors and the activity of specific negative regulators that control the output through TLRs, are responsible for the control of intestinal cell homeostasis [7]–[12]. Misregulation of these negative regulatory control mechanisms leads to intestinal inflammatory pathology.

Certain enteropathogens are able to stimulate intestinal inflammation [13]. For example, *Salmonella enterica* serovar Typhimurium (*S.* Typhimurium), a major cause of food-borne illness, causes intestinal inflammation leading to diarrhea, which is essential for its replication and spread [14],[15]. The mechanisms by which these or other intestinal pathogens bypass the negative regulatory controls in the intestinal epithelium to induce inflammatory responses are poorly understood. Although limited gene expression studies have shown that *S.* Typhimurium reprograms gene expression in cultured cells leading to the production of proinflammatory cytokines [16]–[18], it is unclear to what extent these responses overlap with responses stimulated through the activation of innate immune receptors. The ability of *S.* Typhimurium to stimulate intestinal responses requires the activity of a type III protein secretion system (TTSS) encoded within its pathogenicity island 1 (SPI-1) [16]. However, it is unclear whether this system stimulates inflammatory responses directly, or whether it works in conjunction with surface innate immune receptors that may recognize components of this machine, or cytoplasmic receptors that may recognize conserved bacterial products, since this TTSS is also required for *S.* Typhimurium to enter epithelial cells [19]. Here we report that *S.* Typhimurium can stimulate innate immune responses in cultured epithelial cells through the activity of bacterial effector proteins delivered by its TTSS and in a manner that is independent of innate immune receptors. This mechanism has implications for the ability of *S.* typhimurium to induce intestinal inflammation.

## Results

### *Salmonella* Typhimurium induces innate immune responses in cultured epithelial cells

**Figure 1:**
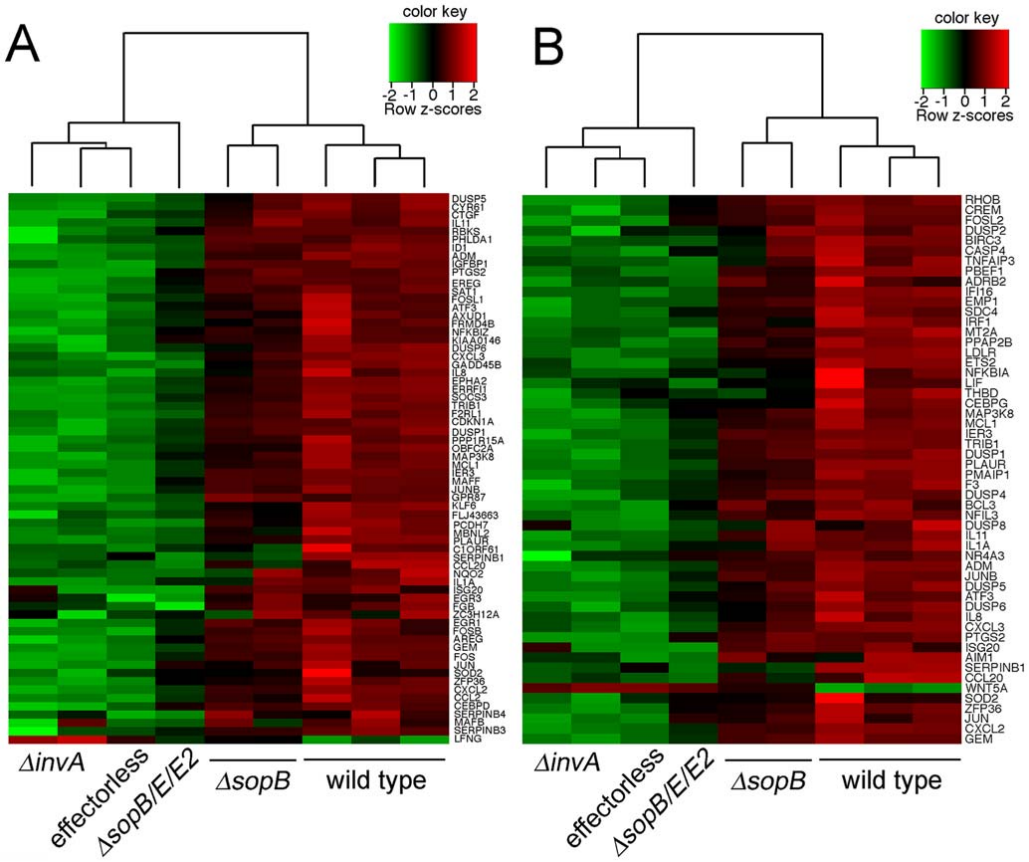
*Salmonella* Typhimurium type III secretion effector proteins induce innate immune responses in epithelial cells. A Cluster analysis of the differentially expressed genes using the ratio of log2-transformed fold change in gene expression values (relative to uninfected cells) in cells infected with the indicated strains of *S.* Typhimurium. Genes and strains were clustered using unsupervised hierarchical clustering with complete linkage, and the data are visualized as row-normalized heat maps as indicated in the color scale. Each vertical row represents an independent experiment. Shown are genes that exhibited at least 4-fold change of expression (over uninfected controls) in all three experiments in cells infected with wild-type *S.* Typhimurium. The full data set are provided in Table S1. B Cluster analysis of differentially expressed genes which exhibited at least 2 fold change of expression (over uninfected controls) in all three experiments in cells infected with wild-type *S.* Typhimurium and that had been previously shown to be part of the pathogen response gene cluster [20]. Genes and strains were clustered using unsupervised hierarchical clustering with complete linkage, and the data are visualized as row-normalized heat maps as indicated in the color scale.

**Figure 2:**
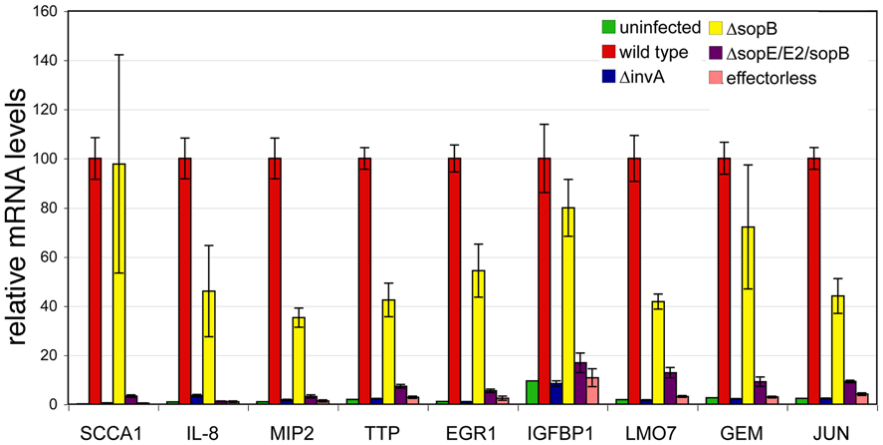
Quantitative RT-PCR analysis of the *Salmonella* Typhimurium-induced transcriptional response. Cultured human epithelial cells were infected with different strains of *S.* Typhimurium (as indicated) and the levels of expression of selected genes were analyzed by qRT-PCR after reverse transcription of RNA samples extracted from cells infected with the indicated *S.* Typhimurium strains. The transcript levels were normalized to the levels of GAPDH in each sample, which were found to remain constant under any of the conditions used in the microarray experiments. Values are expressed as percentage of the fold change observed in cells infected with wild type (considered 100%) and represent the mean±s. e. m. of at least three independent experiments. Fold change for the different reporter genes after infection with wild type was as follows: SCCA1: 749; IL-8: 105; MIP2: 100; TTP: 52; EGR1: 89; IGFBP1: 11; LMO7: 54; GEM: 38; JUN: 42. The differences between the values of cells infected with wild type vs those infected with *ΔinvA*, *ΔsopE/ΔSopE2/ΔsopB*, or effectorless were statistically significant (*p*≥0.002, Student *t* test).

In an effort to understand the mechanisms by which *S.* Typhimurium stimulates intestinal innate immune responses and inflammation, we first determined the global transcriptional response of cultured human Henle-407 epithelial cells infected with wild-type *S.* Typhimurium using DNA microarray analysis. Consistent with previous more limited studies [18], infection of epithelial cells with wild-type *S.* Typhimurium led to a very significant transcriptional reprogramming in comparison to uninfected cells (Fig. 1 and Tables S1 and S2). We identified 66 genes whose expression changed at least 4-fold in cells infected with wild-type *S.* Typhimurium relative to uninfected controls (Fig. 1A) and 290 genes whose expression changed at least 2-fold (Table S1). The majority (235 of 290) of these genes showed increased expression in response to infection and, in some cases, up to several hundred fold (Table S1). We validated the changes of gene expression observed by the microarray analysis by performing quantitative real-time PCR (qRT-PCR) determination of the mRNA (Fig. 2) or in some cases western blot analysis of protein levels (Fig. S1) of selected genes or gene products, respectively. The transcriptional program stimulated by wild-type *S.* Typhimurium infection included several genes whose products are pro-inflammatory such as several chemokines and cytokines and their receptors [e. g. CXCL2 (Mip2a), CXCL3 (Mip2b), IL-8, IL1a, IL11, IL1R1, COX-2, TNFRSF10D, IL4R, TNFRSF12A]. *S.* Typhimurium also induced the stimulation of expression of a number of transcription factors (e. g. FOS, FOSB, FOSL1, JUN, JUNB, EGR1, EGR3, ATF3, STAT3) that amplify the immune response and that most likely are constituents of a positive feed-back loop resulting in the stimulation of expression of themselves and other pro-inflammatory genes. In addition, bacterial infection stimulated the expression of several genes whose products limit the immune response, which most likely constitute a negative feed-back loop to preserve homeostasis and limit damage as a consequence of the inflammatory response. These included the mRNA stability regulator tristetraprolin (ZFP36), the suppressors of cytokine signaling SOCS2 and SOCS3, NAD(P)H dehydrogenase (NQO2), the NF-κB inhibitor IκB zeta, and several members of the DUSP family of tyrosine phosphatases (DUSP1, DUSP2, DUSP4, DUSP5, DUSP6, DUSP8).

The transcriptional response to wild-type *S.* Typhimurium exhibited striking similarities with reported transcription profiles of cells stimulated by agonists of receptors of the innate immune system such as TLRs, or the core response to other microbial pathogens [20]. A gene ontology analysis indicated that genes involved in the innate immune response were significantly over-represented among those induced 2-fold or more by wild-type *S.* Typhimurium infection (p<0.05). In fact, 54 of the 290 differentially-expressed genes belong to the cluster of genes that have been defined as the ‘common host response’ to pathogens (Fig. 1B), a group of genes that are induced in many different cell types in response to exposure to several different microbial pathogens [20]. The proportion is much higher if genes induced by other bacterial pathogens are considered (Table S3). These results indicate that this pathogen has the capacity to stimulate a pro-inflammatory response in cultured epithelial cells that exhibits a great deal of commonality with the innate immune responses triggered by agonists of innate immune receptors or by other pathogens.

### *S.* Typhimurium stimulation of innate immune responses in epithelial cells requires the TTSS effector proteins SopE, SopE2, and SopB

To determine the extent to which the transcriptional changes induced by *S.* Typhimurium depend on the components and activities of its SPI-1 TTSS, we examined the transcriptional response of cultured Henle-407 epithelial cells infected with an isogenic *ΔinvA* mutant strain. InvA is an essential component of the SPI-1 TTSS and therefore the *S.* Typhimurium *ΔinvA* mutant is defective in all phenotypes dependent on this system, which include bacterial internalization into non-phagocytic cells and the induction of programmed cell death in macrophages [21],[22]. The transcriptional profile of cultured epithelial cells infected with this mutant strain closely resembled that of uninfected cells (Fig. 1 and Tables S1 and S2), demonstrating that the transcriptional reprogramming stimulated by wild-type *S.* Typhimurium requires a functional SPI-1 TTSS. The levels of lipopolysaccharide, flagellin, and other potential agonists of innate immune receptors are not affected by the *invA* mutation and are therefore identical to those of wild type (Fig. S2) [21]. Hence, these results also indicate that the responses observed in cells infected with wild-type *S.* Typhimurium cannot be the result of the stimulation of surface Toll-like receptors by these bacterial PAMPS.

The requirement of a functional SPI-1 TTSS for *S.* Typhimurium to trigger transcriptional reprogramming in cultured epithelial cells suggested the possibility that one or more effector proteins delivered by this system are responsible for the stimulation of those responses. Alternatively, such responses could be the result of other activities dependent on a functional TTSS. For example, the outermost component or “needle substructure” of the TTSS needle complex organelle is absent from the *ΔinvA* mutant strain [23],[24]. Therefore, it is possible that the needle substructure itself may be directly recognized by innate immune receptors in epithelial cells thus stimulating transcriptional responses. In addition, delivery of proteins by the TTSSs requires the deployment of a group of TTSS secreted protein translocases, which mediate the passage of effector proteins through the host cell plasma membrane [25],[26]. Therefore, it is possible that potential membrane perturbations caused by the deployment of the TTSS protein translocases, which does not occur in cells infected with the *ΔinvA* mutant, could be sensed by the cell triggering innate immune responses [25],[26]. Finally, stimulation of innate immune responses may be the result of the TTSS-mediate delivery of some agonist of intracellular immune receptors (e. g. flagellin).

To distinguish among these possibilities we examined the transcriptional responses of human epithelial cells infected with a *S.* Typhimurium strain lacking the protein effectors delivered by the SPI-1 TTSS (from here forth referred as “effectorless”). This strain displays a wild-type TTSS organelle, including the needle component, is fully capable of deploying the translocases SipB, SipC and SipD, and it is therefore competent for TTSS-mediated protein translocation [27]. Cells infected with the *S.* Typhimurium effectorless strain showed a transcriptional profile similar to that of uninfected cells or cells infected with the TTSS-defective *invA* mutant (Fig. 1, Fig. 2, and Table S1). These results ruled out the hypothesis that the innate immune responses stimulated by wild-type *S.* typhimurium are the result of the recognition of the needle component of the TTSS by innate immune receptors. In addition, these results excluded the hypothesis that the *S.* typhimurium-induced responses are the result of membrane perturbations induced by the deployment of the translocases, or the delivery of intracellular receptor agonists such as flagellin since all these activities or agonists are present in this mutant strain. Rather, these results unambiguously indicate that one or more effector proteins delivered by this TTSS must be responsible for the induction of the transcriptional reprogramming.

**Figure 3:**
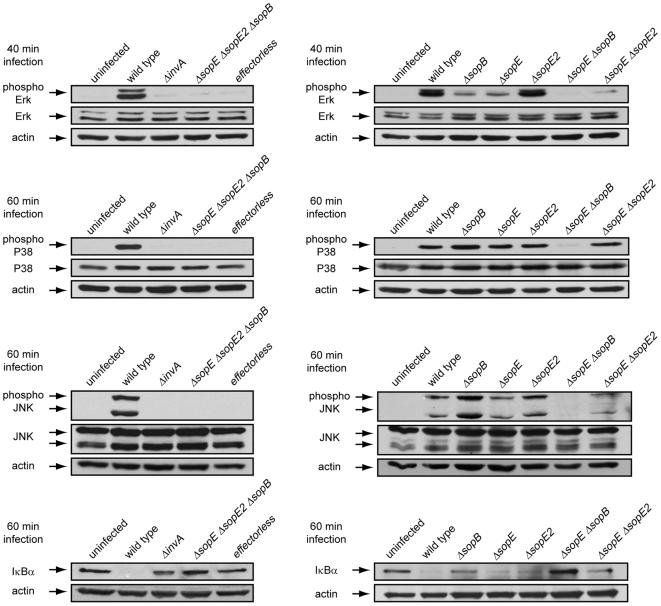
Stimulation of MAP kinases and NF-κB in epithelial cells infected with type III secretion mutants of *Salmonella* Typhimurium. Cultured human epithelial cells were infected with different strains of *S.* Typhimurium and at different times after infection (as indicated), activation of the different kinases was evaluated by western immunoblot using antibodies directed to the phosphorylated (activated) form of the MAP kinases, or to the NF-κB inhibitor IκBα. Equal loading of the different samples was confirmed by re-probing the blots with antibodies directed to actin and/or to the different kinases.

The guanidyl nucleotide exchange factors SopE and SopE2 and the phosphoinositide phosphatase SopB are good candidates to mediate these responses since, in a functionally redundant manner, they activate Rho-family GTPases [28]–[30], which can lead to MAPK and NF-κB activation [31]–[33]. Cells infected with a *ΔsopE ΔsopE2 ΔsopB* triple mutant strain showed a transcriptional profile largely similar to that of uninfected cells or cells infected with a strain lacking a functional SPI-1 TTSS (Fig. 1, Fig. 2, and Tables S1 and S2). Although strains carrying individual mutations in each one of these effectors retained their ability to activate MAPKs and NF-κB (Fig. 3), the Δ*sopE ΔsopE2* Δ*sopB S.* Typhimurium triple mutant strain did not activate these signaling pathways (Fig. 3). These results are consistent with the hypothesis that the activation of these signaling pathways by the bacterially-encoded Rho-family GTPase activators SopE, SopE2, and SopB is central to *S.* Typhimurium's ability to stimulate innate immune responses.

In addition to its ability to activate Rho-family GTPases, SopB mediates activation of AKT by poorly understood mechanisms that require its phosphoinositide phosphatase activity [34]. However, we found that the transcriptome of cells infected with this strain was largely equivalent to that of cells infected with wild-type *S.* Typhimurium (Fig. 1, Fig. 2, and Tables S1 and S2) arguing against a prominent role for AKT in the stimulation of the transcriptional responses. Taken together, these results indicate that SopE, SopE2, and SopB, which operating in a functionally redundant manner activate Rho-family GTPases, MAPK and NF-κB signaling, are responsible for the transcriptional reprogramming induced by *S.* Typhimurium in cultured epithelial cells.

### *Salmonella* Typhimurium stimulation of transcriptional responses in epithelial cells does not require intracellular sensors

**Figure 4:**
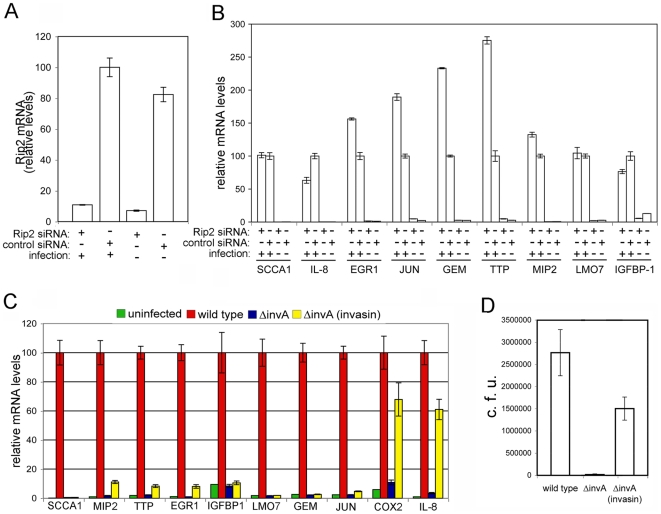
*Salmonella* Typhimurium stimulation of transcriptional responses in epithelial cells does not require the intracellular sensors. A Depletion of Rip2 by RNAi. Cells transfected with siRNAs directed to Rip2 or with an irrelevant control siRNA were infected with wild-type *S.* Typhimurium and the levels of Rip2 in infected and uninfected cells were measured by qRT-PCR. The transcript levels were normalized to the levels of GAPDH. Values are expressed as percentage of the levels of Rip2 transcript in cells that had been treated with a control siRNA, which was considered 100%, and represent the mean±s. e. m. of at least three independent determinations. B Rip2 is not required for *S.* Typhimurium stimulation of gene expression in epithelial cells. Cells in which Rip2 had been depleted by RNAi were infected with the indicated strains of *S.* Typhimurium and the levels of expression of selected genes were analyzed by qRT-PCR after reverse transcription of RNA samples extracted from infected cells. The transcript levels were normalized to the levels of GAPDH. Values are expressed as percentage of the fold change observed in cells that had been treated with a control siRNA and infected with wild-type *S.* Typhimurium, which was considered 100%, and represent the mean±s. e. m. of at least three independent experiments. C *S.* Typhimurium internalized into epithelial cells through a heterologous internalization pathway does not stimulate the transcriptional responses induced by wild type. Cells were infected with a wild-type *S.* Typhimurium, an isogenic *ΔinvA* mutant, or the same mutant expressing *Yersinia pseudotuberculosis* invasin protein (p-invasin). The levels of expression of selected genes were analyzed by qRT-PCR after reverse transcription of RNA samples extracted from infected cells. The transcript levels were normalized to the levels of GAPDH. Values are expressed as percentage of the fold change observed in cells that had been infected with wild-type *S.* Typhimurium, which was considered 100%, and represent the mean±s. e. m. of at least three independent experiments. The differences between the values of cells infected with wild type vs those of uninfected cells or cells infected with the *ΔinvA*, or *ΔinvA* (p-invasin) strains were statistically significant (*p*≥0.001, student *t* test), except for the values corresponding to COX2 and IL-8 in cells infected with the *ΔinvA* (p-invasin) strain. D Levels of intracellular *S.* Typhimurium after internalization through the invasin-mediated pathway. Epithelial cells were infected with wild-type *S.* Typhimurium or the type III secretion-defective *invA* mutant expressing the *Yersinia pseudotuberculosis* invasin protein and the levels of intracellular bacteria were measured by the gentamicin protection assay as indicated in Materials and Methods. Results represent the colony forming units that resisted the gentamicin treatment due to their intracellular location and are the mean±standard deviation of three independent experiments.

The SPI-1-TTSS *S.* Typhimurium mutant strains (i. e. *ΔinvA, ΔsopE/ΔsopE2/ΔsopB*, or “effectorless”), which were unable to stimulate transcriptional responses, posses “wild type” agonists of the innate immune system (i. e. LPS, flagellin, etc.) (Fig. S2). Consequently, these observations suggest that *S.* Typhimurium can trigger innate immune responses in a manner that does not require innate immune receptors such as TLRs. However, since the SPI-1 TTSS, and in particular the effector proteins SopE, SopE2, and SopB, are also required for bacterial internalization, the *ΔsopE/ΔsopE2/ΔsopB* or the “effectorless” mutant strains are unable to enter into host cells [28],[32]. Therefore, it is formally possible that the gene expression changes observed in cells infected with wild type *S.* Typhimurium are stimulated by intracellular bacteria through the intracellular cytoplasmic NLRs such as Nod1 or Nod2 [5], which can sense conserved bacterial products to stimulate innate immune responses [35]. To test this hypothesis we depleted cells of Rip2, a kinase essential for Nod1 and Nod2 signaling [36]. Depletion of Rip2 by RNAi (Fig. 4A), which had no effect on the ability of *S.* Typhimurium to enter epithelial cells (Fig. S3), did not prevent the transcriptional responses stimulated by *S.* typhimurium infection (in fact, the expression of some the reporter genes examined was actually higher in the Rip2-depleted cells) (Fig. 4B). Therefore, these results indicate that the Nod1 or Nod2 receptors are not required for the stimulation of the SPI-1 TTSS-dependent transcriptional responses.

To further address the potential contribution of cytoplasmic sensors to the *S.* Typhimurium SPI-1 TTSS-mediated stimulation of transcriptional responses in epithelial cells we used an alternative approach. We reasoned that if cytoplasmic sensors were responsible for the stimulation of cellular responses by intracellular *S.* Typhimurium, internalization of a SPI-1 TTSS-defective mutant strain by an alternative entry pathway should lead to the stimulation of the same transcriptional responses observed in cells infected with wild type *S.* typhimurium. We therefore expressed in the SPI-1 TTSS-defective Δ*invA* mutant strain the *Yersinia pseudotuberculosis* invasin protein, which mediates bacterial uptake by interacting with α4-β1 integrin receptors [37],[38]. Infection of epithelial cells with the *S.* Typhimurium *ΔinvA* (p-invasin) strain did not stimulate the expression of 7 out of 9 reporter genes tested (Fig. 4C) despite the presence of similar levels of intracellular bacteria to that of cells infected with wild-type *S.* Typhimurium (Fig. 4D). The reduced but significant increase in the expression of IL-8 and COX2 is most likely due the effect of the stimulation of α4-β1 integrins by invasin, since these genes have been reported to be induced by the invasin protein [39]. These results indicate that the intracellular location of *S.* Typhimurium is not sufficient to stimulate the transcriptional responses observed after infection with wild-type *S.* Typhimurium. Furthermore, these observations are consistent with the hypothesis that specific signaling pathways triggered by the activity of the SPI-1 TTSS effectors SopE, SopE2, and SopB are responsible for the stimulation of the transcriptional reprogramming in epithelial cells.

### Cdc42 is required for *Salmonella* Typhimurium stimulation of transcriptional responses in epithelial cells

**Figure 5:**
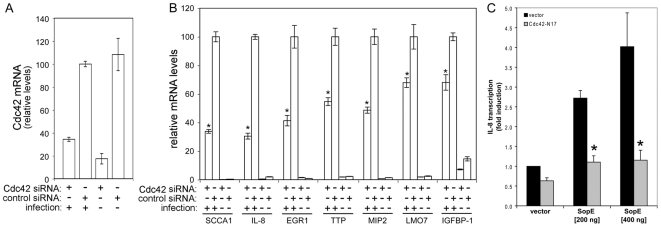
Cdc42 is required for *Salmonella* Typhimurium stimulation of transcriptional responses in epithelial cells. A Depletion of Cdc42 by RNAi. Cells transfected with an siRNA directed to Cdc42 or control cells transfected with an irrelevant construct, were infected with wild-type *S.* Typhimurium and the levels of Cdc42 in infected and uninfected cells were measured by qRT-PCR. The transcript levels were normalized to the levels of GAPDH. Values are expressed as percentage of the levels of Cdc42 transcript in cells that had been treated with a control siRNA and infected with wild-type *S.* Typhimurium, which was considered 100%, and represent the mean±s. e. m. of at least three independent determinations. B Cells in which Cdc42 had been depleted by RNAi were infected with the indicated strains of *S.* Typhimurium and the levels of expression of selected genes were analyzed by qRT-PCR after reverse transcription of RNA samples extracted from infected cells. The transcript levels were normalized to the levels of GAPDH. Values are expressed as percentage of the fold change observed in cells that had been treated with a control siRNA, which was considered 100%, and represent the mean±s. e. m. of at least three independent experiments. * : indicate that values are statistically significantly different (*P*<0.001, Student *t* test) from those of the control infected cells. C Henle-407 cells were co-transfected with an IL-8 transcription firefly-luciferase reporter plasmid along with a plasmid encoding renilla luciferase (to standardize transfection), and when indicated, with the indicated amount of a plasmid encoding SopE, a vector control, or a plasmid encoding a dominant negative mutant form of Cdc42 (Cdc42^N17^). The stimulation of IL-8 transcription in transfected cells was assayed by measuring the levels of firefly luciferase as indicated in Materials and Methods. Values represent fold induction in cells transfected with the SopE plasmid over the value of cells transfected with the plasmid vector alone and are the mean±standard deviation of three independent measurements. * : values statistically significant different from vector control (*P*<0.001, Student *t* test).

SopE, SopE2, and SopB stimulate Rho-family GTPase signaling by different mechanisms. SopE and SopE2 are exchange factors for Rac1, Cdc42, and RhoG [32],[40], while SopB, through its phosphoinositide phosphatase activity, stimulates the RhoG exchange factor SGEF and an unknown exchange factor for Cdc42 [29]. Both Rac1 and RhoG are required for *S.* Typhimurium entry into cells [29],[32]. Cdc42, in contrast, is dispensable for bacterial entry although it is required for efficient stimulation of MAPK [29],[31]. In fact, *S.* Typhimurium can enter cells depleted of Cdc42 in a manner indistinguishable from non-depleted cells [29]. Furthermore, the vesicular traffic and intracellular location of *S.* typhimurium in Cdc42-depleted and non-depleted cells is indistinguishable (Fig. S4). Therefore, the lack of involvement of Cdc42 in the entry and intracellular fate of *S.* Typhimurium allowed us to specifically test its potential role in the transcriptional reprogramming induced by wild-type bacteria, independent from any secondary effect due to actin remodeling or the intracellular location of the internalized bacteria. We found that depletion of Cdc42 significantly prevented the stimulation of expression of the reporter genes (Fig. 5). These results indicate that this Rho-family GTPase plays a critical role in the SPI-1 TTSS-dependent transcriptional reprogramming induced by wild-type *S.* Typhimurium in epithelial cells. The observation that Cdc42 depletion did not completely block the stimulation of gene expression by *S.* Typhimurium suggests that signaling through the other Rho-family GTPases such as Rac1 and RhoG may also contribute to the stimulation of nuclear responses. Alternatively, incomplete depletion of Cdc42 could also account for this result since the transfection efficiency of the cell line used in this study is less than 100%.

We further explored the ability of the *Salmonella* encoded Rho-family GTPase activators to stimulate inflammatory responses by transiently expressing SopE in epithelial cells and examining its effect on IL-8 expression using a transcriptional reporter construct. Transient expression of SopE resulted in a significant increase in IL-8 transcription, and this effect was effectively prevented by the expression of dominant negative Cdc42 (Cdc42^N17^) (Fig. 5C). These results further demonstrate that bacterial effector proteins such as SopE can stimulate an inflammatory response, and that such a response requires Rho-family GTPases.

### *S.* Typhimurium induces intestinal inflammation in mice deficient in innate immunity pathways

**Figure 6:**
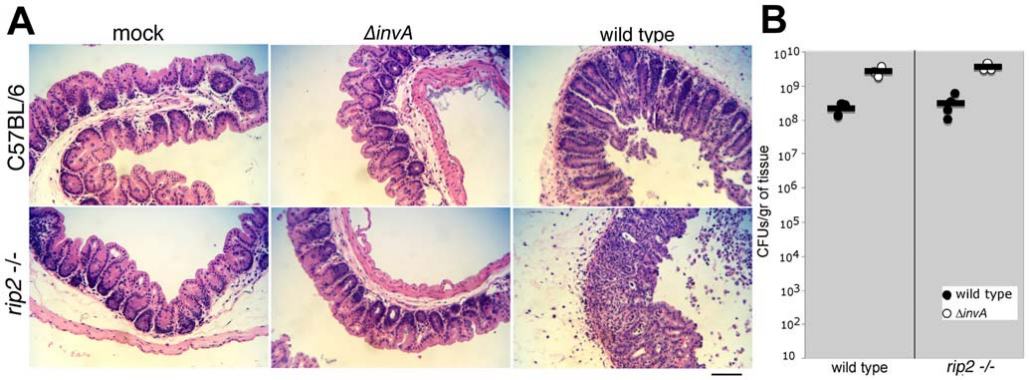
*S.* Typhimurium induces intestinal inflammation in Rip2-deficient mice. A C57BL/6 or *rip2−/−* mice were treated with 20 mg of streptomycin, and 24 hs after antibiotic treatment, mice were either mock infected or infected orally with 10^8^ of wild type *S. typhimurium*, or the isogenic Δ*invA* (type III secretion defective) mutant. Forty-eight hours after infection, ceca were removed, fixed, embedded in paraffin, and tissue sections were stained with hematoxilin and eosin. Bar indicates 100 µm. Similar results were obtained in four independent animals for each group. B Mice were treated and infected with *S. typhimurium* as indicated above and bacterial loads in the ceca were determined 48 hs post-infection.

*S.* Typhimurium can induce colitis in streptomycin-treated Myd88-deficient mice [41] suggesting that signaling through most TLRs is not required to induce inflammation in this infection model. TLR4, the main Toll-like receptor involved in the control of *S.* Typhimurium infection in mice [42], can signal in a Myd88-independent fashion [6]. However, we found that *S.* Typhimurium can induce inflammation in Myd88/TLR4 deficient mice in the same manner as in wild-type animals (Fig. S5). Furthermore, *S.* Typhimurium can also induce inflammation in caspase-1 deficient mice [43]. These results indicate that TLR signaling or activation of the caspase-1 inflammasome [44] are not essential for the inflammatory response to *S.* Typhimurium. To assess the potential contribution of the NLRs Nod1 and Nod2 in *S.* Typhimurium-induced intestinal inflammation, we tested the ability of wild-type *S.* Typhimurium to induce colitis in Rip2-deficient animals. Using the streptomycin-treated mouse model, we found that wild-type *S.* typhimurium induced inflammation in a manner that was indistinguishable from the inflammation observed in wild type animals (Fig. 6). Furthermore, the ability of *S.* Typhimurium to induce inflammation in these animals also required the SPI-1 TTSS since a type III-deficient *ΔinvA* mutant did not cause observable pathology in this infection model system (Fig. 6). Taken together, these results indicate that in this model system, the ability of *S.* Typhimurium to cause inflammation through the activity of its TTSS effectors does not require known receptors of the innate immune system, and are consistent with the results obtained with cultured epithelial cells. Furthermore, these results are also consistent with the observation that SPI-1 TTSS effector proteins are required for the induction of intestinal inflammation in animal models of infection [45]–[48].

## Discussion

Intestinal pathogens such as *S.* Typhimurium must induce intestinal inflammation to secure the acquisition of scarce nutrients as well as their spread to other hosts through the induction of diarrhea [49]. An intact intestinal epithelium does not respond to the multitude of potential agonists of the innate immune system. Such responsiveness would be detrimental in the context of the presence of the abundant intestinal microbial flora containing a plethora of innate immune receptor agonists. Although the mechanisms for this unresponsiveness are not fully understood, it is thought that these mechanisms involve exclusion of innate immune receptors from the epithelial cells' apical side, and the expression of specific negative regulators of innate immune receptor signaling [7]–[12]. Therefore, to induce inflammation, enteropathogens must be able to circumvent these negative regulatory mechanisms [13]. We have shown here that *S.* Typhimurium has evolved a unique mechanism to stimulate innate immune responses in epithelial cells in a manner that is independent of the canonical innate immune receptors and conserved bacterial products or PAMPs. Rather, we have shown that *S.* Typhimurium uses a specific set of effector proteins, SopB, SopE2, and SopE, which are delivered into cells via its SPI-1 TTSS, to activate these responses. We have demonstrated that these responses are not the consequence of bacterial internalization and its subsequent detection by cytoplasmic sensors. Instead, *S.* Typhimurium stimulation of innate immune responses in epithelial cells are the result of the effector-mediated stimulation of Rho-family GTPases leading to the activation of MAPK and NF-κB signaling pathways. Nevertheless, these responses very closely mimic those induced by the stimulation of innate immune receptors such as TLRs or NLRs. It has been previously reported that TLR2 signaling requires Rac-1 [50], presumably at steps downstream from the receptor proximal components of the signaling cascade. Therefore, by targeting similar downstream components of the signaling cascade of innate immune receptors, *S.* Typhimurium effectors can trigger similar outputs as those triggered by the stimulation of those receptors. This mechanism may allow *S.* Typhimurium to bypass the negative regulatory mechanisms that prevent signaling through innate immune receptors in intestinal epithelial cells and thus initiate the inflammatory response at this site.

*S.* Typhimurium can induce intestinal inflammation in mice deficient in various innate immune pathways such as *myd88−/−* [41], caspase 1−/− [43], or as we have shown here, myd88/TLR4−/− and rip2−/− deficient mice. These observations indicate that our observations with cultured epithelial cells are likely to be relevant *in vivo*. Innate immune receptors, however, do play a major role in controlling *S.* Typhimurium infection and spread to systemic tissues, since mice with deficiencies in innate immune pathways are more susceptible to *S.* Typhimurium infection. Innate immune receptor pathways may also contribute to the amplification of the inflammatory response in the gut once initiated by *S.* Typimurium through the specific adaptations described here. However, our results as well as previous *in-vivo* studies [45]–[48] show that with an intact epithelium, *S.* Typhimurium needs its SPI-TTSS to initiate an intestinal inflammatory response presumably via the mechanism reported here.

It is well established that many pathogens have evolved specific adaptations to counteract their hosts' innate immune responses [51],[52]. However, we have shown here that *S.* Typhimurium has evolved specific mechanisms to stimulate innate immune responses, a remarkable adaptation presumably evolved to bypass mechanisms that are in place in intestinal cells to prevent inflammation. Although the mechanisms that prevent intestinal epithelial cells from responding to innate immune receptor agonists are poorly understood, it is well established that misregulation of such mechanisms can lead to chronic inflammatory conditions such as inflammatory bowel disease or Crohn's disease [7]–[11],[53]. The information gained from the study of a pathogen capable of inducing intestinal inflammation could lead to a better understanding of very important but poorly understood chronic inflammatory pathologies and may lead to novel therapeutic strategies.

## Materials and Methods

### Bacterial strains, cell lines and infections

The wild-type strain of *S. enterica* serovar Typhimurium (*S. typhimurium*) SL1344 and its isogenic derivatives used in this study, Δ*invA* (SB136), *ΔsopE* (SB856), *ΔsopE2* (SB1300), Δ*sopB* (SB1120), *ΔsopE ΔsopE2* (SB1301), *ΔsopE ΔsopB* (SB925), Δ*sopE* Δ*sopE2* Δ*sopB* (SB1302), have been previously described [21], [27]–[29]. The strain referred to as “effectorless” (SB1011) has the relevant genotype Δ*sopA* Δ*sopB* Δ*sopD* Δ*sopE* Δ*sopE2* Δ*avrA* Δ*sptP ΔslrP ΔsspH1*, and has also been previously described [27]. The *S.* Typhimurium Δ*invA* strain expressing the *Yersinia pseudotuberculosis* invasin protein was created by transforming the strain SB136 with plasmid pRI207 encoding the *inv* gene from *Yersinia pseudotuberculosis* [37]. All bacterial strains were cultured under conditions that stimulate the expression of the *Salmonella* Typhimurium pathogenicity island-1 encoded TTSS [54].

For bacterial infections, Henle-407 human epithelial cells (80% confluency) were washed twice with DMEM (without serum and antibiotics) and grown in 2 ml of DMEM (without serum and antibiotics) for 16–20 hs. Cells were then washed twice with Hank's buffered salt solution (HBSS) and allowed to equilibrate in HBSS at 37°C for 15 min, and infected at an MOI of 30. In experiments using the *S.* Typhimurium *ΔinvA* mutant strain expressing invasin, an MOI of 100 was used to obtain equal number of internalized bacteria as that of cells infected with wild type. One hour after infection, each well was washed twice with 2 ml of DMEM containing gentamicin (100 µg/ml) and then grown in DMEM containing gentamicin (100 µg/ml) for three additional hours after which the cells were harvested for RNA extraction. The infections for the microarray experiments were performed in 10 cm dishes. All other infections were performed in 6-well dishes.

### Microarray gene expression profiling

Total RNA from infected cells and uninfected control cells was extracted using the RNeasy Midi kit (Qiagen) following manufacturer's instructions. Sample preparation and hybridization to Affymetrix Human Genome U133 Plus 2.0 gene arrays were performed at the Yale University W.M. Keck facility. Briefly, target cDNA generated from each sample was biotinylated, hybridized, and stained as per manufacturer's recommendation using an Affymetrix GeneChip Instrument System. Arrays were scanned on an Affymetrix GeneChip scanner 3000 according to Affymetrix standard protocols (GeneChip Expression Analysis Technical Manual, Affymetrix, 2004). Data was processed using Affymetrix Microarray Suite version 5.0, scaled to a target intensity of 500. Raw and normalized data have been submitted to the GEO database (http://www.ncbi.nlm.nih.gov/geo/), accession number (pending). Data were further analyzed using GeneSpring (Silicongenetics) and the BioConductor software package [55]. Fold change were calculated for each strain relative to the uninfected control in each experimental group. Genes with a MAS5 change call other than “No Change” were considered to be differentially expressed if they also met a minimum fold change requirement in all the experiments using that strain. Analyzed expression data are presented in Table S1. The gene ontology analysis was performed using the GOstats package by BioConductor [56] with the gene universe consisting of all genes with a MAS5 change call other than “No Change” in any experiment of any mutant strain.

### Gene silencing by RNAi

Depletion of endogenous Cdc42 was performed using a short hairpin sequence targeting human Cdc42 as described previously [29]. Silencing of RIP2K gene expression was achieved using synthetic SMARTpools (Dharmacon), each comprising four proprietary siRNA sequences. Both the RIP2 SMARTpool and short hairpin RNA constructs were transfected into Henle-407 cells using Lipofectamine 2000 (Invitrogen), either alone or in combination with pLZRS-CFP (kindly provided by Walther Mothes) for detection of transfected cells during microscopy. Transfections were allowed to proceed for 48 hrs before serum starvation was begun for subsequent infection experiments. The silencing efficiency of the RNAi constructs was measured by qRT-PCR at the end of the infection experiments as described below, or by western blot analysis using a mouse anti-Cdc42 (BD Biosciences).

### Colocalization of endocytic markers and fluid endocytic tracers with the Salmonella-containing vacuole

The acquisition of LAMP1 by the *Salmonella*-containing vacuole and its co-localization with the endocytic tracer Alexa-Fluor-488-labeled dextran were assayed in Cdc42-depleted and control cells by flurorescence microscopy as previously described [57]. Briefly, Cdc42-depleted and control cells grown on glass coverslips were incubated in the presence of 250 µg/ml dextran-Alexa-Fluor-488 MW 10,000 (Molecular Probes), which was chased into lysosomes as previously described [58]. Three hours before infection, cells were washed twice with PBS and subsequently incubated in cultured medium. Cells were infected for 1 h with a multiplicity of infection of 10 with wild-type *S.* Typhimurium expressing dsRed under the control of an arabinose-inducible promoter [59], which had been grown in the presence of 0.1% arabinose. After additional 3 h in medium containing 100 µg/ml Gentamicin, cells were fixed with 3% PFA and subsequently stained with mouse-anti-LAMP1 (clone H4A3) and goat-anti-mouse-Alexa-Fluor-488 (Molecular Probes). For quantification, the localization of bacteria relative to LAMP1 or dextran was determined in CFP positive cells (to identify transfected cells) using fluorescence microscopy.

### Quantitative real-time PCR

Total RNA was isolated from infected Henle-407 cells using TRIzol reagent (Invitrogen) following the manufacturer's protocol. The final pellet was resuspended in RNAse-free water and further purified using the RNeasy Mini kit (Qiagen), digested with DNAse I (Invitrogen) and used as a template for cDNA synthesis using the iScript reverse transcriptase (Bio-Rad). The cDNA was subject to qRT-PCR using iQ SYBR Green Supermix (Bio-Rad). Reactions were run and measurements were obtained using an iCycler realt time PCR machine and iCycler iQ software (Bio-Rad).

### Kinase activation assays and detection of upregulated proteins

Cells were lysed in lysis buffer containing 10 mM Tris-HCl, pH 7.5, 40 mM Na-Pyrophosphate, 5 mM EDTA, 150 mM NaCl, 1% NP-40, 0.5% Na-Deoxycholate, 0.025% SDS, 1 mM Na-orthovanadate and protease inhibitors (complete Protease Inhibitor Cocktail, Roche). Cell lysates were separated by SDS-PAGE, and examined by western immunoblotting using the following antibodies: rabbit-anti-Erk, rabbit-anti-IκBα, rabbit-anti-JNK/SAPK, mouse-anti-phospho-Erk [Thr 202, Tyr 204], mouse-anti-phospho-IκBα [Ser32, Ser 36], mouse-anti-phospho-JNK/SAPK [Thr 183, Tyr 185], rabbit anti-phospho HSP27 [Ser82] (to evaluate p38 activation), and mouse-anti-phospho-P38 [Thr 180, Tyr 182], all purchased from Cell Signaling Technology (Danvers, MA), rabbit-anti-P38, mouse anti SCCA-1, and rabbit-anti-actin purchased from Santa Cruz Biotechnology (Santa Cruz, CA), and rabbit anti Tristetraprolin (TTP) purchased from Abcom (Cambridge, MA).

### IL-8 transcription reporter assay

Cells were transfected with a plasmid encoding SopE [32] along with the reporter plasmid PSB2805, which encodes a fusion between the IL-8 promoter and firefly luciferase [29]. To standardize transfection experiments, cells were also transfected with a plasmid pSB2806, which is a derivative of pCDN3.1 encoding Renilla luciferase [29]. Two days after transfection (including overnight serum starvation), cells were lysed and the levels of firefly and Renilla luciferase were determined using the Dual Luciferase Reporter assay (Promega) according to the manufacturer's instructions. Transfection efficiency was normalized by the comparison of IL-8–induced firefly luciferase levels with that of constitutively expressed Renilla luciferase. Induction of the IL-8 reporter by SopE was expressed relative to that of a vector control.

### Western immunoblot analyis of LPS and flagelin

Levels of LPS and flagellin in whole cell bacterial lysates of the different strains was determined by standard western immunoblot analysis using specific antisera (Difco).

### Animal infection experiments histopathology analysis

Bacterial infections of streptomycin-treated animals and histopathology analysis of tissues were performed essentially as described previously [43]. All animals were maintained and animal experiments conducted in accordance with the guidelines of the Yale Institutional Animal Use and Care Committee.

## Supporting Information

Figure S1 Western blot analysis of the levels of selected proteins whose expression is induced in cultured intestinal epithelial cells infected with wild type *S.* Typhimurium. Cultured intestinal epithelial cells were infected with either wild type or Δ*invA* (type III secretion deficient) *S.* typhimurium strains and at the indicated times after infection, the levels of either SCCA1 or TTP in cell lysates were analyzed by western blotting.
(3.30 MB TIF)

Figure S2 LPS and flagellin levels in wild type and TTSS isogenic mutants of *S.* Typhimurium. The indicated amounts of cultures of the indicated strains were lysed and the LPS and flagellin contents in the whole cell lysates were analyzed by western immunoblot using specific antisera.
(1.23 MB TIF)

Figure S3 Effect of Rip2 depletion on the ability of *S.* typhimurium to invade cultured intestinal epithelial cells. Cells in which Rip2 had been depleted by RNAi or treated with a control RNAi were infected with wild-type S. Typhimurium and the levels of internalized bacteria at the indicated times after infection were determined by the gentamicin protection assay as described in Materials and Methods. (A) The levels of Rip2 mRNA in cells transfected with siRNAs directed to Rip2 or with an irrelevant control siRNA were measured by qRT-PCR after reverse transcription of RNA samples extracted from infected and uninfected cells. The transcript levels were normalized to the levels of GAPDH. Values are expressed as percentage of the fold change observed in uninfected cells that had been treated with a control siRNA, which was considered 100%. (B) The levels of intracellular bacteria represent the number of c. f. u. that survived the gentamicin treatment and are the mean±standard deviation of three independent measurements.
(0.44 MB TIF)

Figure S4 The characteristics of the *S.* typhimurium-containing vacuole in Cdc42-depleted cells are indistinguishable from those in wild type cells. (A) Depletion of Cdc42 does not affect acquisition of LAMP1 by the SCV. Cdc42-depleted and controlled cultured Henle-407 epithelial cells were infected with wild-type *S.* Typhimurium expressing dsRed as indicated in Materials and Methods. Cells were fixed, stained with anti *S.* Typhimurium (red) and an anti LAMP1 (green) antibodies and acquisition of LAMP1 by the SCV was assessed by fluorescence microscopy. Results are the means and standard deviation of three independent experiments, in which at least 300 vacuoles were counted. (B) Depletion of Cdc42 does not affect the accessibility of the SCV to an endocytic tracer. Cdc42-depleted and controlled cultured Henle-407 epithelial cells were labeled with the endocytic tracer Alexa-Fluor-488, which was chased to lysosomes, and infected with wild-type *S.* Typhimurium expressing dsRed as indicated in Materials and Methods. Co-localization of *S.* Typhimurium with the endocytic tracer was determined by fluorescence microscopy. Results are the means and standard deviation of three independent experiments, in which at least 300 vacuoles were counted. (C) Depletion of Cdc42 by RNAi. Cdc42 was depleted from Henle 407 cells as indicated in Materials and Methods and the levels of Cdc42 in treated and control cells was examined by western blot analysis with an anti-Cdc42 antibody.
(2.16 MB TIF)

Figure S5 *S.* Typhimurium induces intestinal inflammation in Myd88/TLR4-deficient mice. C57BL/6 or *myd88*−/− *tlr4*−/− mice were treated with 20 mg of streptomycin, and 24 hs after antibiotic treatment, mice were either mock infected or infected orally with 10^8^ of wild type *S.* typhimurium, or the isogenic Δ*invA* (type III secretion defective) mutant. Forty-eight hours after infection, ceca were removed, fixed, embedded in paraffin, and tissue sections were stained with hematoxilin and eosin. Bar indicates 100 µm. Similar results were obtained in three independent animals for each group.
(9.33 MB TIF)

Table S1 Microarray analysis of the transcriptional responses induced by different strains of *Salmonella* Typhimurium.
(0.08 MB PDF)

Table S2 *P* values (two-tailed *t* test) of the fold changes listed in Table S1.
(0.07 MB PDF)

Table S3 List of genes stimulated by *S.* typhimurium (2 fold or more) that were also stimulated by other pathogens or agonists of the innate immune system.
(0.05 MB PDF)
