# Supplementary figures and images for: Salmonella Typhimurium Type III Secretion Effectors Stimulate Innate Immune Responses in Cultured Epithelial Cells
Source: PLoS Pathog. 2009 Aug 7;5(8):e1000538. doi: 10.1371/journal.ppat.1000538 (PMC2714975; doi:10.1371/journal.ppat.1000538)

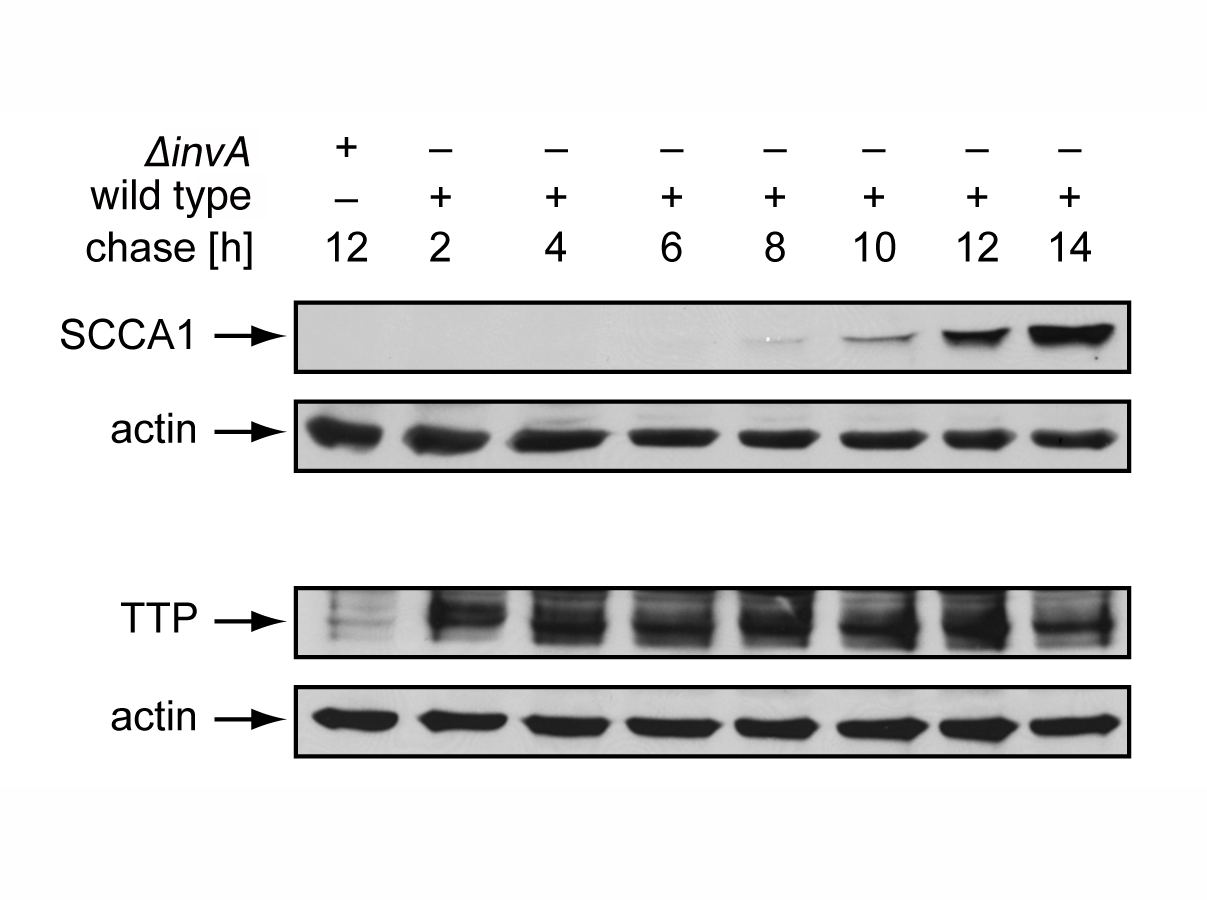

Supplement: Figure S1 — Western blot analysis of the levels of selected proteins whose expression is induced in cultured intestinal epithelial cells infected with wild type S. Typhimurium. Cultured intestinal epithelial cells were infected with either wild type or ΔinvA (type III secretion deficient) S. typhimurium strains and at the indicated times after infection, the levels of either SCCA1 or TTP in cell lysates were analyzed by western blotting. (3.30 MB TIF) [file ppat.1000538.s001.tif]

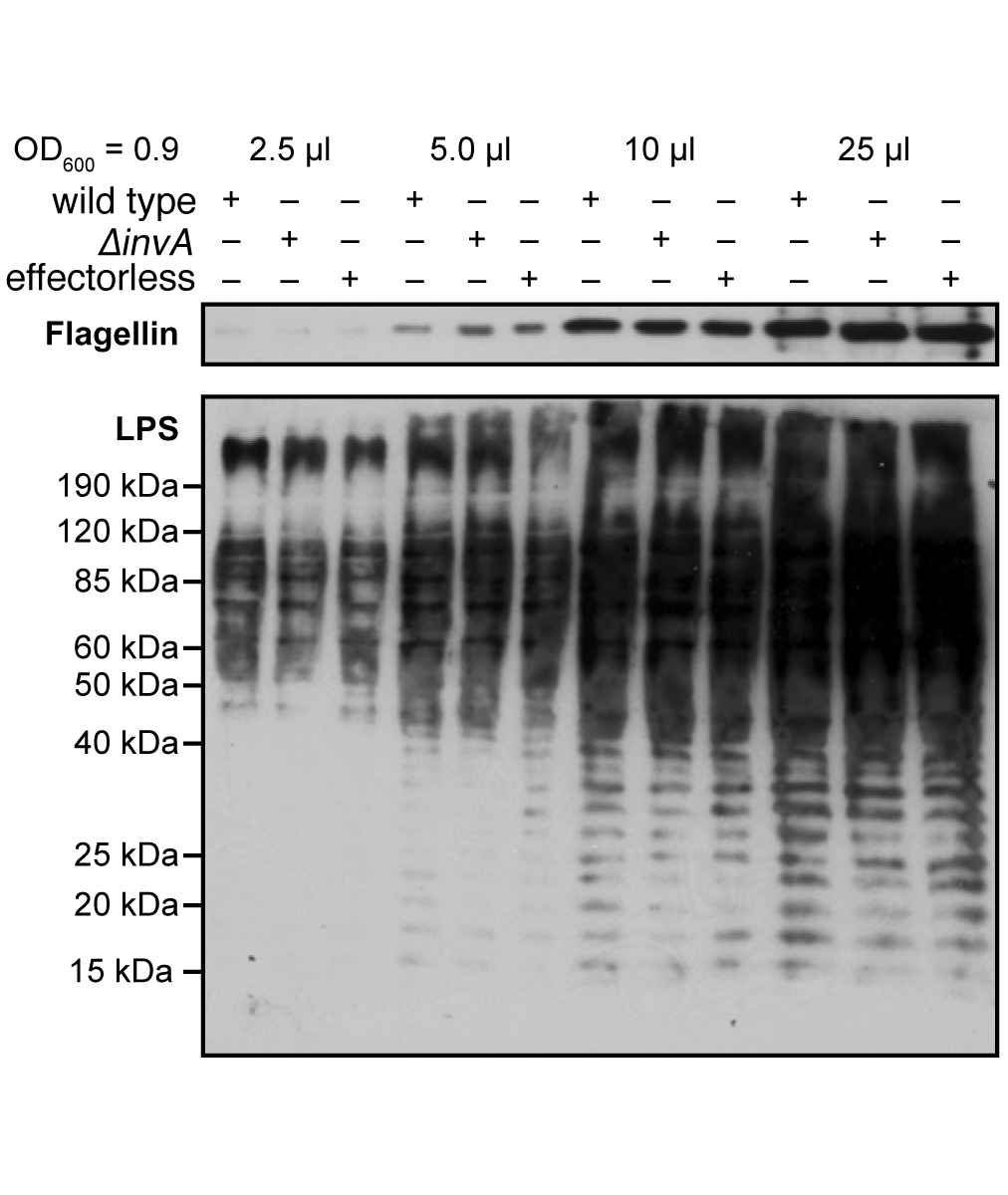

Supplement: Figure S2 — LPS and flagellin levels in wild type and TTSS isogenic mutants of S. Typhimurium. The indicated amounts of cultures of the indicated strains were lysed and the LPS and flagellin contents in the whole cell lysates were analyzed by western immunoblot using specific antisera. (1.23 MB TIF) [file ppat.1000538.s002.tif]

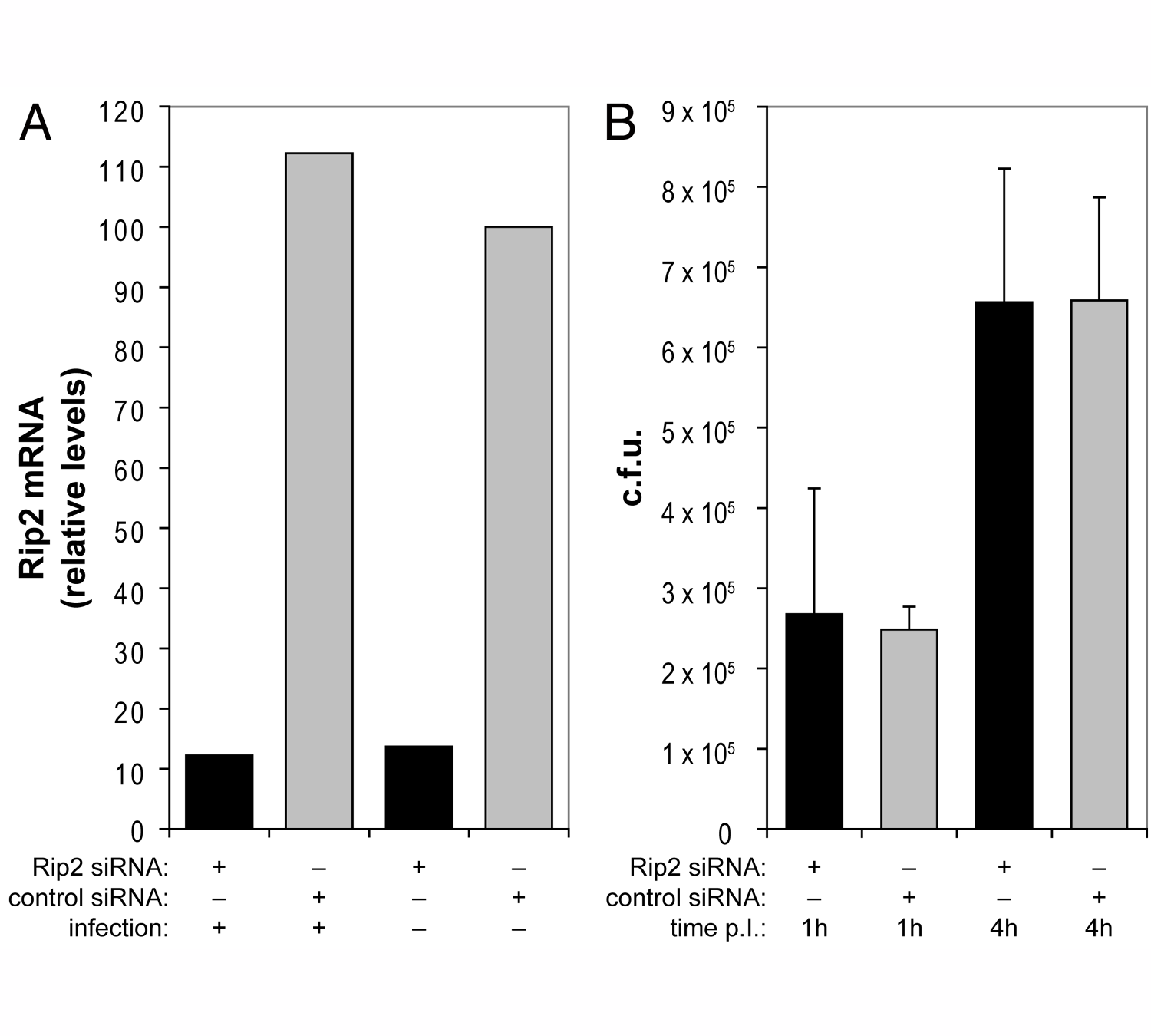

Supplement: Figure S3 — Effect of Rip2 depletion on the ability of S. typhimurium to invade cultured intestinal epithelial cells. Cells in which Rip2 had been depleted by RNAi or treated with a control RNAi were infected with wild-type S. Typhimurium and the levels of internalized bacteria at the indicated times after infection were determined by the gentamicin protection assay as described in Materials and Methods. (A) The levels of Rip2 mRNA in cells transfected with siRNAs directed to Rip2 or with an irrelevant control siRNA were measured by qRT-PCR after reverse transcription of RNA samples extracted from infected and uninfected cells. The transcript levels were normalized to the levels of GAPDH. Values are expressed as percentage of the fold change observed in uninfected cells that had been treated with a control siRNA, which was considered 100%. (B) The levels of intracellular bacteria represent the number of c. f. u. that survived the gentamicin treatment and are the mean±standard deviation of three independent measurements. (0.44 MB TIF) [file ppat.1000538.s003.tif]

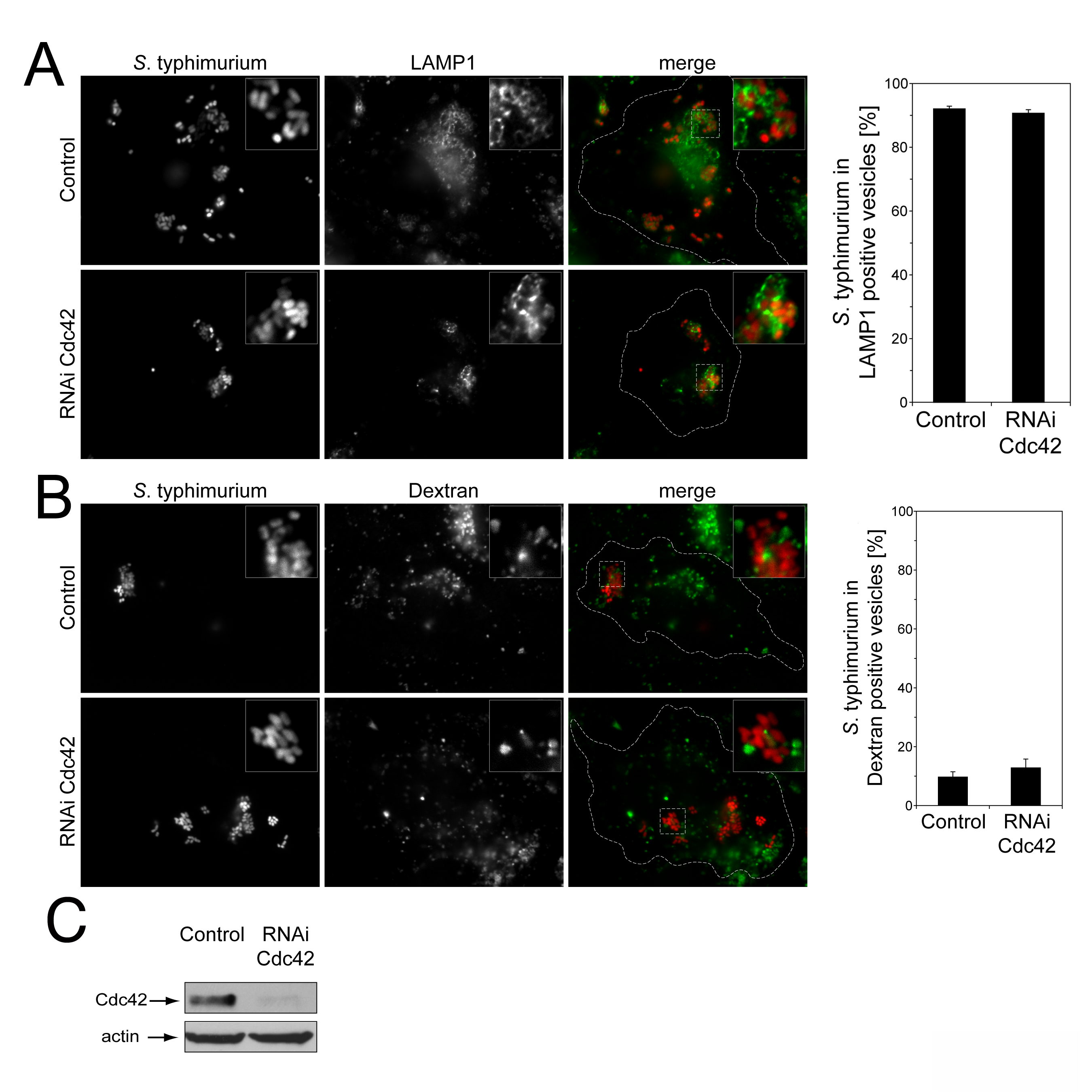

Supplement: Figure S4 — The characteristics of the S. typhimurium-containing vacuole in Cdc42-depleted cells are indistinguishable from those in wild type cells. (A) Depletion of Cdc42 does not affect acquisition of LAMP1 by the SCV. Cdc42-depleted and controlled cultured Henle-407 epithelial cells were infected with wild-type S. Typhimurium expressing dsRed as indicated in Materials and Methods. Cells were fixed, stained with anti S. Typhimurium (red) and an anti LAMP1 (green) antibodies and acquisition of LAMP1 by the SCV was assessed by fluorescence microscopy. Results are the means and standard deviation of three independent experiments, in which at least 300 vacuoles were counted. (B) Depletion of Cdc42 does not affect the accessibility of the SCV to an endocytic tracer. Cdc42-depleted and controlled cultured Henle-407 epithelial cells were labeled with the endocytic tracer Alexa-Fluor-488, which was chased to lysosomes, and infected with wild-type S. Typhimurium expressing dsRed as indicated in Materials and Methods. Co-localization of S. Typhimurium with the endocytic tracer was determined by fluorescence microscopy. Results are the means and standard deviation of three independent experiments, in which at least 300 vacuoles were counted. (C) Depletion of Cdc42 by RNAi. Cdc42 was depleted from Henle 407 cells as indicated in Materials and Methods and the levels of Cdc42 in treated and control cells was examined by western blot analysis with an anti-Cdc42 antibody. (2.16 MB TIF) [file ppat.1000538.s004.tif]

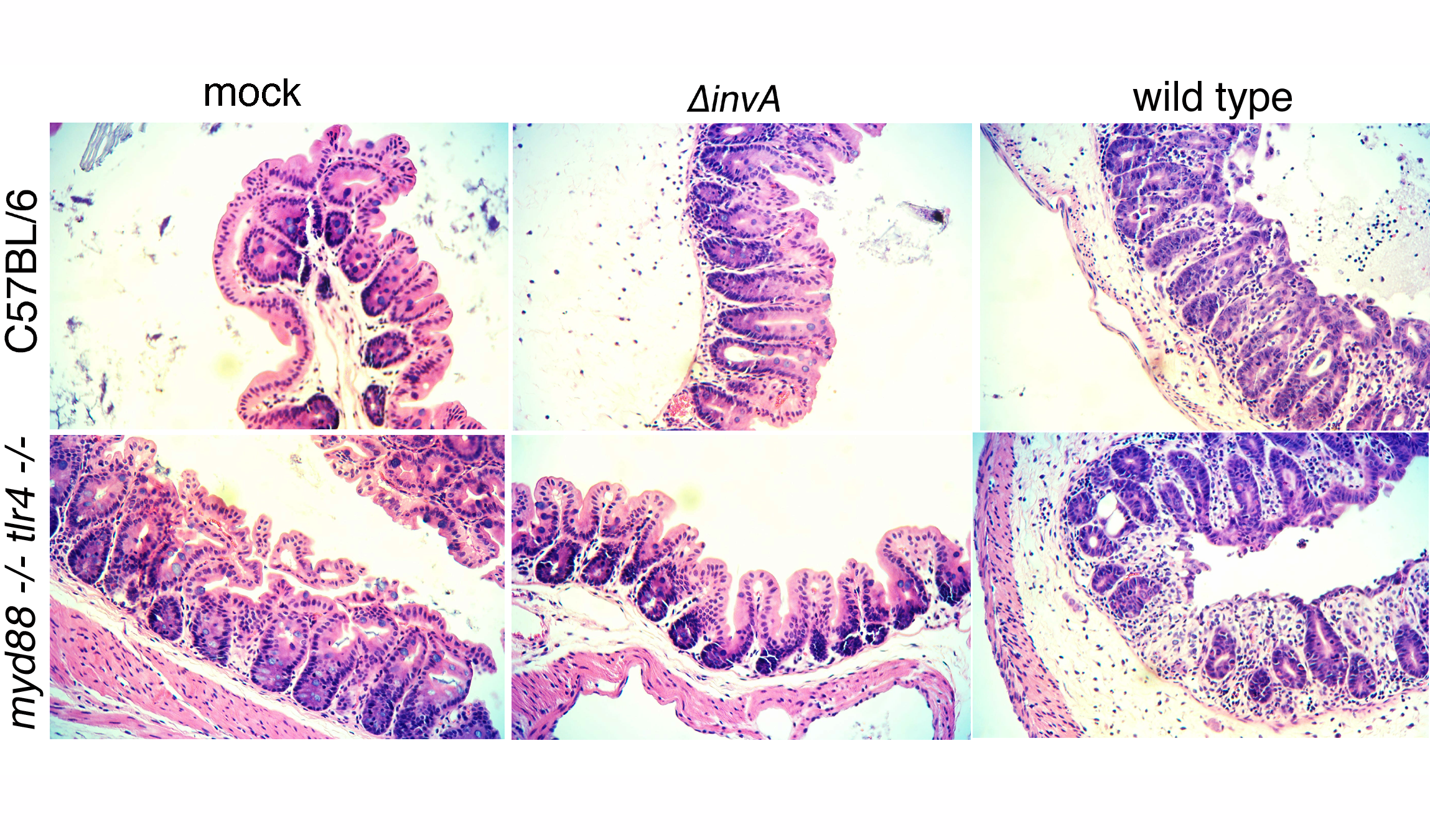

Supplement: Figure S5 — S. Typhimurium induces intestinal inflammation in Myd88/TLR4-deficient mice. C57BL/6 or myd88−/− tlr4−/− mice were treated with 20 mg of streptomycin, and 24 hs after antibiotic treatment, mice were either mock infected or infected orally with 108 of wild type S. typhimurium, or the isogenic ΔinvA (type III secretion defective) mutant. Forty-eight hours after infection, ceca were removed, fixed, embedded in paraffin, and tissue sections were stained with hematoxilin and eosin. Bar indicates 100 µm. Similar results were obtained in three independent animals for each group. (9.33 MB TIF) [file ppat.1000538.s005.tif]
