# Supplementary material for: Salmonella Typhimurium Type III Secretion Effectors Stimulate Innate Immune Responses in Cultured Epithelial Cells
Source: PLoS Pathog. 2009 Aug 7;5(8):e1000538. doi: 10.1371/journal.ppat.1000538 (PMC2714975; doi:10.1371/journal.ppat.1000538)
Supplement: Table S1 — Microarray analysis of the transcriptional responses induced by different strains of Salmonella Typhimurium. (0.08 MB PDF) [file ppat.1000538.s006.pdf]

Table S1: Microarray analysis of the transcriptional responses induced by different strains of *Salmonella* Typhimurium *

Probeset	Gene	ΔinvA	ΔinvA	effectorless	ΔsopB/E/E2	ΔsopB	ΔsopB	wild type	wild type	wild type
206411_s_at	ABL2	1.0	0.8	1.1	1.2	1.7	2.1	4.6	3.2	2.5
231907_at	ABL2	0.9	1.0	1.1	1.4	2.0	1.7	3.0	2.5	2.1
202912_at	ADM	0.8	0.9	1.3	1.7	5.3	5.3	11.3	9.2	9.2
206170_at	ADRB2	0.8	1.1	1.2	1.0	2.8	2.1	4.9	2.6	2.1
212543_at	AIM1	2.0	2.0	1.1	1.4	5.7	3.2	2.5	7.5	7.0
210517_s_at	AKAP12	0.8	1.0	1.6	1.2	1.6	1.7	4.0	2.3	2.3
227529_s_at	AKAP12	0.8	0.7	1.2	0.9	1.4	2.0	2.5	2.0	2.0
227530_at	AKAP12	0.9	0.9	1.4	1.0	1.7	1.9	3.7	2.5	2.8
227337_at	ANKRD37	0.7	1.1	1.1	1.3	2.1	2.3	4.9	2.8	2.5
219496_at	ANKRD57	0.8	0.9	1.2	1.7	2.3	2.5	4.6	3.7	4.3
227034_at	ANKRD57	0.9	0.9	1.1	1.5	2.5	2.6	4.3	3.7	3.7
205239_at	AREG	0.4	0.9	1.3	4.9	12.1	14.9	24.3	16.0	17.1
220382_s_at	ARHGAP28	1.0	1.3	0.6	1.1	0.9	0.8	0.5	0.4	0.5
242727_at	ARL5B	1.4	0.9	0.9	1.5	2.1	1.7	2.3	2.0	2.0
224797_at	ARRDC3	0.9	0.8	1.0	1.2	2.5	2.3	3.7	3.5	3.2
202672_s_at	ATF3	0.8	0.8	1.1	2.6	3.7	6.1	17.1	6.5	7.0
1554980_a_at	ATF3	1.7	1.1	0.8	1.5	1.7	2.6	5.7	2.6	3.7
225557_at	AXUD1	1.0	0.6	1.2	2.3	5.3	4.0	17.1	8.6	6.5
225612_s_at	B3GNT5	0.9	1.0	1.1	1.5	2.1	2.3	3.5	2.8	2.6
1554835_a_at	B3GNT5	1.1	1.3	1.3	1.2	2.8	2.3	3.2	2.8	3.2
221485_at	B4GALT5	1.1	1.1	1.0	1.3	1.9	1.5	2.3	2.1	2.3
204032_at	BCAR3	1.0	1.0	1.1	1.5	1.4	1.7	3.0	2.1	2.5
1557257_at	BCL10	0.9	1.2	2.3	2.3	3.5	2.0	9.2	2.8	2.0
204908_s_at	BCL3	1.1	1.1	1.0	2.0	3.7	2.5	3.7	4.3	2.8
201169_s_at	BHLHB2	1.1	0.9	0.9	1.1	1.9	2.5	3.7	3.7	4.0
201170_s_at	BHLHB2	0.8	0.7	0.9	1.1	1.7	2.1	4.0	3.2	3.5
210538_s_at	BIRC3	1.1	1.4	1.5	1.4	1.9	3.0	4.0	3.2	3.2
220178_at	C19ORF28	0.9	1.2	1.4	1.7	2.6	1.7	3.0	3.0	2.1
1558834_s_at	C1ORF62	1.6	1.6	1.3	2.0	2.5	1.7	8.6	4.3	4.0
219474_at	C3ORF52	0.7	0.9	1.5	2.5	2.1	1.9	4.3	3.2	2.3
1553868_a_at	C5ORF36	1.1	0.7	0.8	0.5	0.4	0.7	0.3	0.4	0.4
218541_s_at	C8ORF4	0.4	1.5	0.7	0.5	3.0	3.0	3.2	3.7	3.7
213596_at	CASP4	1.1	1.1	0.9	1.7	1.5	3.2	4.6	2.8	3.0
216598_s_at	CCL2	1.1	1.1	0.7	3.7	21.1	12.1	207.9	55.7	39.4
205476_at	CCL20	0.9	1.4	0.9	0.7	4.6	1.5	4.9	18.4	17.1
202769_at	CCNG2	1.1	0.9	0.7	0.9	0.9	0.7	0.5	0.4	0.3
220046_s_at	CCNL1	0.9	1.0	0.9	1.1	1.9	1.5	3.5	2.1	2.3
1555411_a_at	CCNL1	1.0	1.0	1.1	1.1	1.9	1.9	3.5	2.5	2.5
1555827_at	CCNL1	1.6	1.1	1.2	1.2	2.5	2.3	3.7	3.5	2.5
204695_at	CDC25A	0.9	1.0	1.3	1.9	1.6	1.9	2.5	2.1	2.0
1555772_a_at	CDC25A	1.0	0.9	1.1	1.4	1.9	2.1	2.8	2.0	2.5
204677_at	CDH5	1.2	1.1	1.1	1.3	1.5	2.6	2.5	2.8	2.6
202284_s_at	CDKN1A	1.1	0.8	1.1	1.6	3.7	4.0	4.0	4.9	5.3
203973_s_at	CEBPD	0.9	1.2	1.1	2.8	6.5	5.3	8.6	8.6	7.5
213006_at	CEBPD	0.1	0.2	0.5	3.7	8.6	6.1	13.0	14.9	8.0
204203_at	CEBPG	0.9	1.0	1.2	1.4	1.4	1.4	2.8	2.0	2.1
219270_at	CHAC1	0.2	1.1	1.1	0.9	1.7	2.1	2.3	4.6	4.0
228297_at	CNN3	1.1	0.9	0.9	0.9	2.0	1.7	2.3	2.3	2.3
207630_s_at	CREM	0.9	0.7	1.1	1.7	2.1	2.5	3.2	2.6	2.6
209967_s_at	CREM	0.9	0.8	1.1	1.6	2.0	2.1	3.0	2.5	3.0
214508_x_at	CREM	1.1	0.8	1.1	1.5	1.9	1.7	2.8	2.6	2.0
209101_at	CTGF	0.6	0.6	1.4	1.6	3.5	7.0	7.0	4.9	7.5
209774_x_at	CXCL2	0.7	1.1	1.4	4.0	24.3	22.6	84.4	64.0	52.0
1569203_at	CXCL2	4.6	6.1	3.5	1.0	39.4	10.6	21.1	24.3	10.6
207850_at	CXCL3	1.5	1.0	0.7	1.3	4.9	7.5	11.3	13.0	13.0
212977_at	CXCR7	1.0	0.9	0.8	0.8	0.4	0.4	0.4	0.3	0.4
201289_at	CYR61	0.6	0.6	1.1	1.3	2.8	4.6	6.5	4.9	7.5
210764_s_at	CYR61	0.9	0.8	1.1	1.3	3.2	5.3	8.0	5.3	8.6
231919_at	DBT	1.1	0.9	0.9	0.7	0.5	0.6	0.5	0.4	0.5
204602_at	DKK1	0.7	0.8	1.5	1.7	4.6	4.9	4.6	3.5	4.0
1557394_at	DLGAP4	1.9	1.7	1.0	1.2	1.9	1.7	2.3	2.1	3.5
231297_at	DOT1L	1.0	1.1	1.1	1.1	2.1	2.0	2.0	2.8	2.1
201041_s_at	DUSP1	0.7	0.7	1.1	1.7	3.0	4.0	3.5	3.7	4.6
201044_x_at	DUSP1	0.9	0.7	1.0	1.6	3.2	4.0	4.6	4.3	6.1

204794_at	DUSP2	1.1	0.8	1.5	1.4	1.6	2.8	2.6	2.1	2.6
204014_at	DUSP4	0.9	0.9	1.3	1.6	2.6	3.5	4.3	3.5	3.2
204015_s_at	DUSP4	0.8	0.9	1.2	1.4	2.3	2.5	3.0	2.5	2.5
209457_at	DUSP5	1.1	1.0	1.1	1.7	3.5	6.5	8.6	6.5	9.8
208891_at	DUSP6	0.6	0.6	1.1	0.9	2.8	2.8	3.5	5.3	4.6
208892_s_at	DUSP6	0.8	0.9	1.2	1.1	2.8	3.7	4.9	6.1	6.5
208893_s_at	DUSP6	0.9	0.4	1.1	1.9	2.8	5.7	14.9	10.6	13.9
206374_at	DUSP8	2.5	0.9	0.9	1.3	1.6	4.9	2.3	3.2	7.0
201693_s_at	EGR1	1.5	1.1	1.1	1.0	11.3	13.0	34.3	17.1	10.6
201694_s_at	EGR1	0.7	1.4	1.3	3.5	18.4	11.3	42.2	24.3	11.3
227404_s_at	EGR1	1.0	1.1	1.5	4.0	17.1	18.4	34.3	18.4	18.4
206115_at	EGR3	3.5	1.7	0.4	0.8	3.7	8.6	4.0	4.0	10.6
218696_at	EIF2AK3	1.0	1.0	1.1	1.3	2.1	2.1	3.5	2.8	2.5
201303_at	EIF4A3	1.1	1.1	1.1	1.2	2.5	1.9	2.5	2.5	2.3
235592_at	ELL2	0.5	0.9	1.1	1.2	1.9	1.7	3.0	2.6	2.3
201324_at	EMP1	0.8	1.1	1.1	1.1	2.1	2.3	3.0	2.6	2.6
201325_s_at	EMP1	1.0	0.8	1.1	1.1	2.1	2.1	3.0	2.3	2.8
242868_at	EPAS1	0.5	1.6	1.1	3.5	4.9	2.5	12.1	6.1	3.0
203499_at	EPHA2	1.0	0.9	1.1	1.7	3.2	3.7	5.7	5.3	6.1
205767_at	EREG	0.9	1.0	1.5	3.7	8.0	9.2	9.2	8.6	9.8
224657_at	ERRFI1	0.8	0.9	1.1	1.5	3.0	4.0	6.5	6.1	6.5
224454_at	ETNK1	0.9	1.4	1.4	1.6	2.3	1.9	3.2	3.0	2.0
1555355_a_at	ETS1	11.3	1.9	1.7	2.5	1.4	17.1	3.5	2.3	16.0
201328_at	ETS2	1.1	0.8	0.7	1.1	1.5	1.5	3.2	2.6	2.6
201329_s_at	ETS2	0.9	0.8	0.8	1.2	1.6	1.6	2.8	2.5	2.5
213506_at	F2RL1	1.0	0.8	0.9	1.1	3.5	3.7	7.5	4.3	5.3
204363_at	F3	0.7	1.0	1.2	1.5	2.5	2.8	3.5	3.5	4.6
227410_at	FAM43A	0.8	0.8	1.0	1.0	0.4	0.6	0.5	0.3	0.4
232202_at	FAM83B	1.0	1.0	1.1	1.3	2.1	2.0	3.0	2.8	3.0
223240_at	FBXO8	1.0	0.8	0.9	1.0	0.8	0.8	0.5	0.4	0.5
205650_s_at	FGA	0.8	0.7	0.9	2.5	3.7	2.1	13.9	5.7	3.2
204988_at	FGB	1.6	1.2	0.6	0.1	5.3	13.9	4.3	9.2	16.0
204421_s_at	FGF2	1.0	1.1	1.1	1.0	1.5	1.9	2.3	2.1	2.3
226621_at	FGG	1.1	0.9	1.1	1.5	2.0	1.9	2.1	2.3	2.5
243309_at	FLJ27352	1.1	0.9	1.1	2.0	4.0	3.7	7.5	4.6	3.7
229521_at	FLJ36031	1.5	0.8	1.0	1.0	1.9	1.6	4.6	2.0	2.3
239331_at	FLJ43663	0.4	1.3	1.1	1.4	3.5	2.5	9.2	7.0	5.7
219250_s_at	FLRT3	0.9	0.8	1.0	1.6	1.3	2.3	3.5	2.5	2.6
222853_at	FLRT3	0.9	0.9	1.1	1.3	1.6	1.5	2.8	2.5	2.5
1559060_a_at	FNIP1	0.7	1.1	0.6	0.8	2.0	2.5	7.0	3.0	2.5
209189_at	FOS	0.4	0.5	0.9	3.2	14.9	13.0	52.0	27.9	24.3
202768_at	FOSB	1.1	1.1	0.7	3.0	10.6	12.1	39.4	16.0	9.8
204420_at	FOSL1	0.9	0.9	1.7	2.3	4.6	6.1	13.9	7.0	6.1
218880_at	FOSL2	1.1	1.2	1.1	2.0	2.1	2.3	3.0	2.5	2.5
225262_at	FOSL2	1.1	0.9	1.1	1.9	1.6	2.0	2.6	2.1	2.5
227475_at	FOXQ1	1.1	1.1	1.1	1.4	1.9	1.9	4.9	2.8	2.8
213056_at	FRMD4B	1.1	1.1	1.9	2.8	3.7	6.1	21.1	7.5	9.2
210220_at	FZD2	1.5	1.0	0.8	0.7	0.5	0.5	0.4	0.3	0.4
207574_s_at	GADD45B	0.9	0.8	1.0	0.9	2.3	2.6	9.2	5.7	5.7
209304_x_at	GADD45B	0.8	0.9	0.9	0.7	2.3	2.1	6.5	4.6	5.3
209305_s_at	GADD45B	0.8	1.1	0.9	0.9	2.5	1.5	7.5	4.6	4.0
213560_at	GADD45B	1.1	1.1	1.1	0.9	3.2	6.1	17.1	12.1	14.9
204457_s_at	GAS1	1.1	0.8	0.8	0.6	0.4	0.4	0.3	0.3	0.3
219508_at	GCNT3	1.1	1.1	1.2	1.2	1.9	2.1	2.8	3.2	2.3
221577_x_at	GDF15	1.0	0.9	0.9	2.6	3.5	4.3	3.2	5.7	4.9
204472_at	GEM	1.1	0.8	1.3	2.8	16.0	21.1	59.7	27.9	27.9
205100_at	GFPT2	1.4	1.4	1.1	1.1	2.1	1.5	2.6	3.5	2.3
204222_s_at	GLIPR1	0.7	1.0	1.4	1.1	1.6	1.4	6.5	3.2	3.0
226136_at	GLIPR1	1.1	0.9	0.9	0.6	1.4	1.7	3.2	3.0	2.0
226142_at	GLIPR1	0.8	0.8	2.1	1.7	1.2	1.5	3.7	2.1	2.6
223079_s_at	GLS	1.1	1.0	1.2	1.6	2.3	2.6	2.6	2.5	2.6
219936_s_at	GPR87	1.0	1.0	1.4	1.7	6.1	5.3	4.0	4.9	5.3
203108_at	GPRC5A	0.9	0.9	1.3	1.4	2.5	2.6	2.1	2.3	2.6
232290_at	GPRC5A	0.8	1.2	1.7	2.5	3.0	1.7	5.3	3.7	2.6
218706_s_at	GRAMD3	0.9	1.0	1.0	1.1	1.7	1.9	2.0	2.0	2.0
212434_at	GRPEL1	1.0	1.0	1.1	1.6	1.6	1.7	2.3	2.0	2.0
223541_at	HAS3	1.6	0.4	3.0	3.0	1.4	2.3	4.6	2.3	3.7

1557169_x_at	HCG11	0.9	1.1	1.1	1.2	0.7	0.6	0.5	0.3	0.5
232026_at	HERC4	0.9	1.1	1.6	1.2	1.7	2.6	3.0	2.0	2.3
203394_s_at	HES1	0.9	1.2	1.3	1.6	3.5	3.0	9.8	4.0	3.0
218839_at	HEY1	1.1	1.0	0.8	0.5	0.2	0.2	0.4	0.3	0.3
202934_at	HK2	0.9	1.0	1.1	1.5	1.7	1.6	2.8	2.1	2.0
205579_at	HRH1	0.9	1.1	1.5	1.6	2.5	2.3	4.6	2.8	2.8
221667_s_at	HSPB8	1.1	0.9	1.2	1.1	1.5	1.9	3.5	2.3	2.1
208937_s_at	ID1	0.9	1.0	1.1	1.6	7.5	6.5	9.2	13.9	10.6
201631_s_at	IER3	0.8	1.2	1.1	1.9	4.0	3.7	4.9	4.9	4.6
218611_at	IER5	0.9	1.1	1.1	1.3	2.1	2.3	3.7	2.5	2.6
206332_s_at	IFI16	1.1	1.1	0.9	1.1	2.0	2.0	3.0	2.8	3.0
208966_x_at	IFI16	1.1	1.1	1.0	1.1	2.1	2.0	3.0	2.8	2.8
205302_at	IGFBP1	1.3	1.1	0.9	1.7	7.5	7.0	7.0	9.2	8.0
206924_at	IL11	0.5	1.0	0.9	1.4	5.3	13.0	8.0	6.1	12.1
210118_s_at	IL1A	0.5	0.5	0.7	1.5	1.5	6.5	4.9	5.3	9.8
202948_at	IL1R1	1.5	0.8	1.0	1.2	3.0	3.5	3.7	4.3	4.9
203233_at	IL4R	1.0	0.9	1.4	1.3	1.7	1.9	2.5	2.0	2.3
202859_x_at	IL8	1.6	1.3	0.8	1.7	3.7	9.2	36.8	9.2	16.0
211506_s_at	IL8	2.0	1.1	0.9	1.0	2.8	6.5	11.3	7.5	21.1
1557174_a_at	IRAK1BP1	0.9	0.9	0.7	0.7	0.6	0.5	0.4	0.5	0.5
202531_at	IRF1	1.0	1.1	1.1	1.2	1.9	1.5	3.0	2.6	2.3
204698_at	ISG20	4.6	1.1	1.1	1.0	5.7	7.0	6.1	8.6	4.3
219361_s_at	ISG20L1	0.8	0.9	1.1	2.0	2.5	2.8	3.0	2.8	2.5
205032_at	ITGA2	1.0	1.0	1.4	0.9	1.1	2.0	3.2	2.3	3.5
227314_at	ITGA2	0.9	0.8	0.9	0.8	1.5	2.1	4.0	3.0	4.3
213146_at	JMJD3	0.6	1.4	1.4	1.4	3.2	1.5	5.3	4.6	2.6
212723_at	JMJD6	1.0	1.1	1.1	1.5	1.7	1.7	2.6	2.1	2.1
226352_at	JMY	0.9	1.0	1.0	1.1	0.8	0.7	0.5	0.5	0.4
243446_at	JUB	1.1	2.8	1.6	1.7	5.7	4.3	3.2	9.8	10.6
201464_x_at	JUN	1.0	1.2	1.2	2.6	8.0	10.6	34.3	17.1	21.1
201465_s_at	JUN	0.7	1.2	1.0	12.1	9.2	19.7	84.4	11.3	29.9
201466_s_at	JUN	0.9	0.9	0.9	2.6	5.3	4.3	24.3	11.3	12.1
213281_at	JUN	0.5	1.1	1.0	2.0	2.5	5.7	11.3	4.3	7.0
201473_at	JUNB	1.2	0.9	1.1	2.5	5.7	4.9	9.8	7.0	6.5
228325_at	KIAA0146	0.7	0.4	1.1	4.3	5.3	5.3	14.9	8.0	8.0
202386_s_at	KIAA0430	0.9	1.0	1.0	0.9	0.6	0.5	0.4	0.4	0.4
225582_at	KIAA1754	0.8	1.0	1.1	1.5	2.6	2.1	3.2	3.0	2.5
225193_at	KIAA1967	0.8	1.0	0.9	0.7	0.4	0.5	0.5	0.5	0.4
219371_s_at	KLF2	1.1	0.8	0.8	0.8	1.2	1.5	4.3	2.1	2.1
209212_s_at	KLF5	1.1	1.1	1.1	1.6	2.6	2.8	2.6	2.6	2.8
208960_s_at	KLF6	1.5	0.3	1.3	1.4	3.0	5.7	11.3	7.0	12.1
208961_s_at	KLF6	1.1	1.3	1.1	1.6	3.7	3.2	12.1	8.6	9.8
224606_at	KLF6	0.7	0.8	0.8	1.5	3.5	3.7	12.1	8.6	9.2
1555832_s_at	KLF6	0.6	0.7	0.9	1.2	3.5	3.5	12.1	7.5	8.6
226158_at	KLHL24	1.0	1.1	1.1	1.3	0.6	0.6	0.4	0.3	0.2
205157_s_at	KRT17	1.0	1.0	1.5	2.0	2.6	2.6	3.7	3.2	3.5
212236_x_at	KRT17	0.9	1.1	1.4	1.9	2.6	2.3	3.7	3.0	3.0
202067_s_at	LDLR	1.0	0.8	0.9	0.9	1.5	1.9	2.3	2.5	3.2
202068_s_at	LDLR	0.8	0.7	0.8	0.9	1.6	1.7	2.6	2.6	2.8
217173_s_at	LDLR	0.8	0.7	1.1	1.1	1.5	1.1	2.6	2.5	2.0
207170_s_at	LETMD1	1.1	1.1	0.9	0.9	0.7	0.7	0.5	0.5	0.5
242424_at	LETMD1	0.4	0.9	0.9	0.7	0.4	0.4	0.4	0.4	0.1
228762_at	LFNG	1.1	1.6	0.6	0.3	0.4	0.4	0.1	0.3	0.1
214045_at	LIAS	0.8	1.0	0.9	0.8	0.5	0.6	0.4	0.4	0.5
205266_at	LIF	0.9	1.2	1.3	0.9	1.5	1.4	4.3	2.0	2.0
241140_at	LMO7	0.5	1.9	1.0	1.0	2.3	1.5	4.6	2.1	2.1
1556377_s_at	LMO7	0.1	11.3	1.4	3.5	19.7	1.7	8.0	32.0	2.0
231828_at	LOC253039	1.1	0.9	0.8	0.7	0.5	0.5	0.5	0.5	0.5
236076_at	LOC257396	1.0	0.8	0.6	0.4	0.6	0.6	0.2	0.4	0.1
235151_at	LOC283357	0.9	1.1	0.7	0.8	0.7	0.6	0.4	0.3	0.4
1556064_at	LOC284926	0.8	0.7	0.8	0.5	0.4	0.4	0.4	0.3	0.3
222662_at	LOC286044	1.1	1.0	1.0	1.2	1.3	1.2	4.9	3.5	2.1
227940_at	LOC339803	0.9	0.8	0.7	0.6	0.5	0.3	0.4	0.5	0.4
243009_at	LOC441242	0.8	0.9	0.9	0.5	0.3	0.5	0.5	0.4	0.4
226977_at	LOC492311	0.9	0.9	0.8	0.8	0.5	0.5	0.3	0.4	0.4
241874_at	LOC492311	0.9	1.1	0.9	0.7	0.5	0.7	0.3	0.5	0.4
220770_s_at	LOC63920	1.1	1.0	0.9	0.7	0.7	0.5	0.3	0.5	0.4

242396_at	LOC644192	0.6	1.4	1.0	0.4	0.4	0.1	0.4	0.5	0.1
1555978_s_at	LOC727918	0.9	1.2	1.1	0.8	1.5	1.6	2.0	2.0	2.0
217122_s_at	LOC728661	1.0	1.0	0.9	0.8	0.5	0.6	0.5	0.5	0.5
239343_at	LOC728705	0.9	0.9	0.9	0.6	0.4	0.4	0.4	0.4	0.4
228536_at	LOC90826	0.9	1.1	1.4	1.9	3.2	2.1	3.7	2.8	2.3
223533_at	LRRC8C	1.1	1.1	1.2	1.5	2.6	2.5	3.0	2.5	2.3
218559_s_at	MAFB	0.1	8.6	0.5	0.6	9.8	0.9	5.7	18.4	4.3
205193_at	MAFF	1.7	0.9	1.7	3.5	4.9	7.5	8.6	6.5	6.5
36711_at	MAFF	0.9	1.2	1.3	3.2	6.1	5.7	8.0	7.0	7.0
206750_at	MAFK	1.2	1.2	1.1	1.7	1.7	2.1	3.5	2.3	2.0
226206_at	MAFK	0.9	0.9	1.2	2.5	3.5	3.0	4.6	3.7	3.2
224480_s_at	MAG1	0.9	0.9	0.9	1.1	2.1	2.5	2.0	2.3	2.6
205027_s_at	MAP3K8	0.9	0.8	1.1	2.1	2.8	3.7	6.5	4.0	4.9
235421_at	MAP3K8	1.3	1.5	0.9	0.9	1.9	2.3	2.8	2.0	3.5
236067_at	MBNL2	0.8	1.0	1.3	1.1	1.9	1.5	4.9	3.0	2.6
236699_at	MBNL2	0.9	1.1	0.9	1.1	2.5	1.7	7.5	4.9	4.3
200796_s_at	MCL1	1.1	1.1	1.1	1.7	2.8	3.0	5.3	4.0	4.3
200797_s_at	MCL1	1.0	1.1	1.1	1.6	2.5	2.5	3.5	2.8	3.2
200798_x_at	MCL1	0.9	1.0	1.2	1.6	2.5	2.5	3.7	3.0	3.5
214056_at	MCL1	0.8	1.1	1.1	1.4	2.5	3.0	5.7	3.5	4.0
214057_at	MCL1	1.0	1.1	1.3	1.6	2.0	2.5	3.0	2.8	3.0
227175_at	MCL1	1.2	1.1	0.8	1.1	1.7	1.9	2.5	2.3	2.5
212830_at	MEGF9	0.8	0.8	0.9	0.9	0.7	0.7	0.5	0.4	0.4
214696_at	MGC14376	0.9	0.9	1.3	2.0	2.1	2.5	6.1	3.2	3.0
221477_s_at	MGC5618	1.5	1.7	1.4	2.1	2.8	2.5	3.2	3.2	2.8
239549_at	M-RIP	0.6	1.0	1.1	1.0	2.0	1.6	3.0	2.3	2.1
1570588_at	M-RIP	0.8	1.7	0.9	1.5	2.0	1.6	3.0	3.2	2.5
217165_x_at	MT1F	1.0	1.2	1.1	1.0	1.9	1.9	2.0	2.1	2.6
211456_x_at	MT1P2	1.0	1.2	1.2	1.1	1.6	1.7	2.1	2.3	2.6
204326_x_at	MT1X	1.1	1.1	1.1	1.1	1.5	1.9	2.1	2.0	2.5
208581_x_at	MT1X	1.0	1.1	1.2	1.1	1.6	2.0	2.5	2.3	2.8
212185_x_at	MT2A	1.1	1.2	1.1	1.0	1.7	1.9	2.1	2.3	2.6
225344_at	NCOA7	1.1	1.1	0.9	1.4	2.0	1.9	2.1	2.3	2.3
203574_at	NFIL3	1.0	0.8	0.7	1.5	3.0	2.6	5.3	4.6	3.7
201502_s_at	NFKBIA	1.2	1.1	1.1	1.5	1.3	1.6	4.6	2.1	2.6
223217_s_at	NFKBIZ	1.4	1.0	1.5	2.6	4.0	6.1	18.4	5.7	8.6
223218_s_at	NFKBIZ	0.8	1.1	1.5	3.7	5.7	4.9	18.4	7.5	7.0
202585_s_at	NFX1	1.1	0.9	0.8	0.8	0.7	0.6	0.3	0.5	0.5
202237_at	NNMT	1.0	1.0	0.9	1.3	2.8	2.0	2.5	3.0	2.8
202238_s_at	NNMT	0.9	1.1	1.1	1.5	3.0	2.1	2.5	3.2	2.6
231798_at	NOG	0.9	0.9	0.8	0.9	0.4	0.6	0.5	0.4	0.4
244855_at	NQO2	1.1	1.0	0.8	0.9	1.2	13.0	5.3	7.5	19.7
202340_x_at	NR4A1	1.0	1.1	1.1	1.7	3.2	3.0	6.5	3.5	3.2
204621_s_at	NR4A2	0.8	0.7	0.9	1.6	2.6	3.0	9.2	3.0	3.0
235739_at	NR4A2	0.9	1.2	1.1	1.2	2.1	2.6	8.6	3.2	2.6
207978_s_at	NR4A3	0.8	0.7	0.9	1.3	2.1	2.1	4.0	2.8	2.5
209959_at	NR4A3	0.1	0.9	0.9	2.0	2.5	3.2	5.3	3.0	4.3
204435_at	NUPL1	0.7	1.2	1.3	1.5	2.3	1.5	3.2	3.0	2.0
219334_s_at	OBFC2A	0.4	0.9	1.1	1.1	1.6	2.6	3.7	3.5	4.9
222872_x_at	OBFC2A	0.9	0.9	1.0	1.3	2.8	4.0	5.7	4.9	6.5
233085_s_at	OBFC2A	0.7	1.1	1.0	1.7	2.8	2.8	7.0	4.9	5.7
205729_at	OSMR	1.1	0.9	1.1	1.5	1.7	1.7	2.1	2.3	2.3
226140_s_at	OTUD1	1.3	1.1	1.0	1.9	2.1	1.9	4.6	2.3	2.5
243296_at	PBEF1	0.9	1.1	0.8	0.9	2.5	2.1	4.0	3.7	4.0
205534_at	PCDH7	1.1	1.3	0.9	1.0	3.2	2.5	5.3	5.7	4.3
205535_s_at	PCDH7	0.7	1.3	1.1	1.2	1.9	1.6	6.1	3.5	2.8
232382_s_at	PCMTD1	1.1	1.1	1.0	1.0	0.8	0.9	0.5	0.4	0.4
238902_at	PCMTD1	0.9	1.1	1.1	0.8	0.5	0.4	0.4	0.3	0.3
230109_at	PDE7B	0.8	0.8	0.9	0.8	0.5	0.6	0.4	0.4	0.5
202464_s_at	PFKFB3	0.9	1.1	1.1	1.2	1.1	1.5	4.0	2.3	2.3
217996_at	PHLDA1	0.7	1.0	1.3	1.3	4.0	3.7	4.0	5.3	4.6
217997_at	PHLDA1	0.6	0.9	1.5	1.2	2.8	3.5	3.5	3.7	4.6
218000_s_at	PHLDA1	0.4	1.1	1.3	1.3	5.7	5.7	4.6	7.0	6.1
225842_at	PHLDA1	0.8	1.0	1.3	1.1	2.0	2.5	2.6	2.6	2.3
239102_s_at	PICALM	0.8	1.3	1.5	1.3	2.3	1.4	4.3	2.8	2.1
210845_s_at	PLAUR	1.0	0.9	1.1	1.2	2.5	2.5	4.0	4.0	4.0
211924_s_at	PLAUR	1.3	0.9	1.3	1.2	2.0	2.8	3.2	4.3	4.6

1558836_at	PLGLB1	1.0	1.3	1.1	0.9	1.9	1.3	3.7	2.3	2.8
204285_s_at	PMAIP1	0.9	0.9	1.0	1.3	2.0	2.3	4.0	3.2	3.7
204286_s_at	PMAIP1	0.8	0.8	0.9	1.2	2.3	2.3	3.7	3.2	4.0
1554609_at	POLD3	0.9	0.8	0.6	0.6	0.3	0.3	0.4	0.5	0.3
212226_s_at	PPAP2B	0.9	1.0	0.8	0.9	1.9	1.6	2.8	2.3	2.5
212230_at	PPAP2B	1.0	0.9	0.9	1.0	1.5	1.7	2.3	2.3	2.6
218273_s_at	PPM2C	1.1	1.0	0.9	1.1	2.0	1.9	3.5	2.6	2.5
222572_at	PPM2C	1.0	1.0	1.1	1.1	2.0	2.0	3.2	3.0	2.5
202014_at	PPP1R15A	0.8	1.1	1.3	1.7	3.5	3.5	8.0	4.3	4.9
37028_at	PPP1R15A	1.1	1.2	1.0	1.3	2.8	2.6	8.6	4.6	3.2
224692_at	PPP1R15B	0.9	1.1	1.1	1.6	2.0	2.1	2.6	2.3	2.3
1552670_a_at	PPP1R3B	1.3	1.3	0.9	1.1	1.1	1.1	4.0	3.0	3.7
225066_at	PPP2R2D	0.9	1.6	2.8	1.4	2.0	1.4	7.5	3.7	2.1
201594_s_at	PPP4R1	1.0	0.9	1.1	1.1	1.4	1.6	2.0	2.0	2.0
227510_x_at	PRO1073	0.8	1.7	1.1	1.1	1.9	1.6	6.1	2.8	2.8
209586_s_at	PRUNE	1.1	1.1	1.0	0.9	0.6	0.6	0.5	0.5	0.5
210988_s_at	PRUNE	1.1	0.9	1.0	0.9	0.7	0.5	0.4	0.5	0.4
204748_at	PTGS2	1.3	1.3	1.6	5.3	6.1	9.2	8.6	7.5	8.6
1554997_a_at	PTGS2	1.3	1.3	1.4	5.3	7.5	11.3	9.2	8.6	11.3
208965_s_at	PYHIN1	1.1	1.1	1.0	1.1	2.0	2.0	2.5	2.8	2.8
1557432_at	RASAL2	0.8	1.1	0.9	0.9	1.1	1.5	2.8	2.3	2.8
223467_at	RASD1	1.9	0.6	0.9	1.9	2.1	4.9	4.6	3.5	6.1
1568768_s_at	RBKS	0.5	1.1	1.5	3.0	5.7	6.5	6.5	6.5	6.1
228455_at	RBM15	0.7	1.0	1.1	0.8	0.6	0.5	0.5	0.5	0.4
202388_at	RGS2	0.8	0.9	0.9	0.8	1.6	2.1	4.9	3.5	4.3
212099_at	RHOB	0.9	0.9	1.1	1.6	2.1	2.6	2.8	2.5	2.8
1553962_s_at	RHOB	0.9	0.8	1.1	1.9	2.1	2.3	2.8	2.3	2.3
223168_at	RHOU	1.0	1.0	0.8	0.8	0.6	0.6	0.5	0.5	0.4
229285_at	RNASEL	1.1	1.1	0.9	0.6	0.6	0.4	0.2	0.3	0.5
212724_at	RND3	0.9	0.9	0.9	1.1	1.4	1.5	3.7	2.6	2.6
236114_at	RUNX1	1.0	1.2	0.9	1.0	2.1	2.5	2.6	2.8	2.3
238909_at	S100A10	0.8	0.9	0.9	1.1	2.1	1.9	3.0	2.6	2.8
204268_at	S100A2	1.1	0.9	1.1	1.1	1.6	2.3	3.0	3.0	3.7
228923_at	S100A6	1.2	1.1	1.6	2.0	2.8	2.8	4.9	2.8	2.6
203455_s_at	SAT1	1.1	1.4	1.4	1.9	3.2	3.0	3.2	3.5	3.2
210592_s_at	SAT1	1.2	1.4	1.4	1.9	3.5	3.2	3.7	4.0	4.0
213988_s_at	SAT1	1.2	1.5	1.5	2.3	4.9	3.7	6.1	4.9	4.6
230333_at	SAT1	1.9	2.1	2.5	4.0	7.0	6.5	9.2	7.0	8.6
235147_at	SATB2	0.7	1.2	0.2	0.6	0.6	0.6	0.5	0.3	0.5
202071_at	SDC4	0.9	1.1	1.1	1.5	1.9	1.9	3.0	2.3	2.1
234725_s_at	SEMA4B	1.1	1.0	0.9	0.9	1.6	1.7	3.2	2.6	2.3
228398_at	SENP8	1.3	0.9	0.8	0.8	0.5	0.3	0.4	0.4	0.5
1552684_a_at	SENP8	1.0	1.1	0.8	0.8	0.5	0.5	0.2	0.5	0.5
1558937_s_at	SERF1A	0.4	1.1	0.9	0.7	0.6	0.5	0.3	0.4	0.4
239213_at	SERPINB1	1.2	1.3	2.8	0.9	1.9	1.4	9.2	9.2	9.8
209719_x_at	SERPINB3	1.5	2.5	6.1	6.1	34.3	22.6	68.6	68.6	42.2
209720_s_at	SERPINB3	0.9	26.0	27.9	64.0	274.4	294.1	630.3	1260.7	548.7
210413_x_at	SERPINB4	2.5	2.8	3.2	1.6	24.3	13.0	21.1	52.0	26.0
211906_s_at	SERPINB4	0.5	3.7	0.1	0.3	294.1	17.1	11.3	776.0	36.8
223394_at	SERTAD1	1.2	1.1	1.5	1.4	2.6	3.0	5.3	3.7	3.7
202656_s_at	SERTAD2	1.0	0.9	1.0	1.1	1.9	1.9	2.6	2.5	2.3
202657_s_at	SERTAD2	1.0	1.1	1.1	1.2	2.0	2.0	2.6	2.3	2.1
223196_s_at	SESN2	2.8	1.2	1.1	1.1	0.2	1.9	2.8	2.0	5.3
209260_at	SFN	1.0	1.0	1.1	1.2	2.0	1.6	2.1	2.6	2.3
33323_r_at	SFN	1.0	1.1	1.0	1.1	1.7	1.6	2.1	2.3	2.0
201739_at	SGK	0.8	0.8	1.0	1.3	1.9	2.5	2.5	2.1	2.6
242963_at	SGMS2	0.9	0.9	1.1	1.6	2.8	2.5	3.5	2.5	2.6
1569263_at	SLC16A3	1.5	1.1	0.7	1.2	1.7	2.1	6.5	2.0	3.2
230494_at	SLC20A1	0.7	1.1	1.1	1.1	2.0	2.0	4.6	2.0	2.1
239474_at	SLC6A6	1.1	1.7	1.3	1.2	3.0	1.5	4.0	4.9	2.5
223666_at	SNX5	1.1	1.1	1.1	1.6	2.3	2.0	5.3	2.1	2.1
203372_s_at	SOCS2	1.0	0.8	1.0	0.6	1.6	1.2	3.7	2.8	2.5
203373_at	SOCS2	0.9	0.7	0.9	0.8	1.2	1.6	4.0	2.6	3.2
206359_at	SOCS3	1.1	0.8	1.0	1.7	2.6	2.1	6.1	4.3	4.6
206360_s_at	SOCS3	1.2	0.8	1.1	1.3	1.7	1.5	3.0	2.1	2.1
227697_at	SOCS3	0.9	0.8	0.9	1.7	3.2	3.5	7.0	4.9	5.7
215078_at	SOD2	1.9	1.0	3.5	6.5	7.0	8.0	104.0	9.2	9.8

215223_s_at	SOD2	1.3	1.6	1.5	2.0	2.8	2.8	4.6	3.5	3.5
216841_s_at	SOD2	1.2	1.4	1.3	1.9	2.5	2.1	3.0	2.5	2.5
202935_s_at	SOX9	0.9	0.9	0.9	0.9	2.0	2.3	4.0	2.3	2.8
235680_at	STAT3	0.6	0.8	1.1	1.4	2.1	1.6	2.8	2.6	2.1
243213_at	STAT3	0.8	1.9	1.3	1.3	3.2	1.9	4.9	7.0	2.5
203439_s_at	STC2	0.7	0.8	0.9	0.8	1.4	1.1	2.6	2.3	2.1
209238_at	STX3	0.9	1.1	0.9	1.6	2.3	2.1	3.0	3.0	2.0
204067_at	SUOX	1.1	0.9	1.0	0.8	0.6	0.8	0.5	0.5	0.4
230052_s_at	TA-NFKBH	1.0	1.2	1.3	1.5	2.3	2.0	3.2	2.8	2.3
240206_at	TARS	0.5	0.9	0.9	1.1	1.4	2.1	3.0	2.5	2.5
226625_at	TGFBR3	1.1	1.0	0.9	0.8	0.7	0.6	0.5	0.5	0.5
230380_at	THAP2	0.9	1.1	1.3	2.1	2.5	2.8	4.6	2.8	2.6
203887_s_at	THBD	1.0	1.2	1.5	1.3	1.2	1.5	2.3	2.0	2.3
201107_s_at	THBS1	2.0	0.7	1.3	1.1	1.4	2.0	3.0	4.6	4.3
201109_s_at	THBS1	1.0	1.1	1.2	1.1	2.3	2.0	2.6	2.8	3.0
201110_s_at	THBS1	0.9	1.0	1.1	1.1	2.0	2.0	2.3	2.1	2.6
213352_at	TMCC1	0.8	1.0	1.0	0.7	0.4	0.5	0.4	0.5	0.4
227112_at	TMCC1	0.9	0.8	0.8	0.7	0.5	0.5	0.3	0.4	0.5
213338_at	TMEM158	1.1	1.1	1.0	0.9	1.5	1.4	2.5	2.6	2.1
218113_at	TMEM2	1.0	1.1	1.1	1.7	2.5	2.1	2.6	2.6	2.5
224917_at	TMEM49	0.8	1.1	1.5	1.7	3.5	3.0	2.6	3.2	3.0
231697_s_at	TMEM49	0.8	1.0	1.2	2.0	3.5	3.2	2.6	3.5	4.0
1569003_at	TMEM49	0.9	1.1	1.2	1.6	3.0	2.3	2.0	2.6	2.5
222449_at	TMEPAI	1.0	0.9	1.2	1.3	2.5	2.6	3.2	3.0	2.8
202643_s_at	TNFAIP3	1.1	1.0	1.0	0.8	1.5	2.6	5.7	2.8	4.3
202644_s_at	TNFAIP3	1.1	0.9	1.1	0.9	1.2	2.5	5.7	2.3	3.7
227345_at	TNFRSF10D	1.1	1.0	1.0	1.0	1.3	1.7	3.5	2.3	3.2
218368_s_at	TNFRSF12A	0.8	0.9	1.2	1.4	2.5	2.5	2.0	2.3	2.1
202241_at	TRIB1	0.9	0.9	1.1	1.4	3.2	3.7	5.7	4.9	4.6
1554250_s_at	TRIM73	0.9	1.0	1.0	1.2	1.4	1.0	8.6	2.1	2.3
233771_at	TRIO	2.5	1.3	1.5	1.4	2.3	2.6	4.0	2.8	7.0
204094_s_at	TSC22D2	0.9	1.0	1.1	1.2	1.9	2.0	3.2	2.0	2.1
223282_at	TSHZ1	1.1	1.1	1.0	1.1	0.6	0.5	0.4	0.3	0.4
1554588_a_at	TTC30B	0.9	1.0	0.9	0.6	0.5	0.4	0.3	0.5	0.3
204141_at	TUBB2A	0.9	0.9	1.0	1.1	1.5	1.5	3.5	2.5	2.1
201008_s_at	TXNIP	1.1	1.1	1.0	0.8	0.5	0.4	0.3	0.3	0.3
201009_s_at	TXNIP	1.1	1.1	1.0	0.8	0.5	0.4	0.4	0.3	0.3
201010_s_at	TXNIP	0.9	1.1	1.1	0.8	0.5	0.4	0.4	0.4	0.3
204881_s_at	UGCG	1.1	1.2	1.1	1.6	2.8	2.5	3.2	2.3	2.3
221765_at	UGCG	1.3	0.7	0.8	1.3	2.3	2.3	3.0	2.5	2.0
224967_at	UGCG	1.1	0.9	0.9	1.5	2.3	2.0	2.6	2.3	2.3
238542_at	ULBP2	0.5	0.6	0.8	0.8	1.1	1.5	2.3	2.3	2.3
220370_s_at	USP36	0.9	0.9	1.2	1.6	2.3	1.9	3.7	2.6	2.3
221704_s_at	VPS37B	1.5	1.2	1.6	1.9	1.9	1.7	3.0	2.3	2.6
213425_at	WNT5A	0.9	1.0	1.0	0.9	0.8	0.8	0.4	0.5	0.4
227501_at	WSB1	1.1	1.0	1.0	0.8	1.4	1.4	4.0	2.3	2.3
215150_at	YOD1	1.1	0.9	0.9	1.4	2.0	2.1	3.5	2.8	3.5
227309_at	YOD1	1.0	0.9	1.0	1.3	2.0	2.3	3.7	2.5	2.6
227978_s_at	ZADH2	0.9	1.0	1.1	0.9	0.5	0.6	0.4	0.5	0.5
218810_at	ZC3H12A	6.5	1.1	3.5	5.3	3.0	21.1	13.0	4.9	24.3
231899_at	ZC3H12C	0.9	1.1	1.1	1.6	2.1	2.6	2.1	2.3	2.1
1552283_s_at	ZDHHC11	1.1	1.1	0.9	1.1	1.6	2.1	2.3	2.5	2.1
201531_at	ZFP36	1.6	1.2	1.7	6.5	8.0	9.8	39.4	14.9	19.7
214670_at	ZKSCAN1	1.1	0.9	0.9	0.8	0.5	0.5	0.4	0.4	0.4
1557953_at	ZKSCAN1	1.1	1.0	1.1	0.7	0.4	0.5	0.5	0.4	0.5
222619_at	ZNF281	0.9	1.0	1.0	1.1	1.7	1.9	3.5	2.3	2.5
211064_at	ZNF493	1.0	1.1	1.2	0.8	0.8	0.4	0.5	0.4	0.5
227195_at	ZNF503	0.9	1.0	1.2	1.1	1.7	1.5	3.0	2.5	2.0
235304_at	ZNF573	0.7	0.9	1.0	0.7	0.4	0.5	0.4	0.3	0.5
228005_at	ZXDB	1.0	0.8	0.9	1.0	0.7	0.7	0.5	0.4	0.5

* : Values are fold change relative to uninfected cells
