# Supplementary material for: Salmonella Typhimurium Type III Secretion Effectors Stimulate Innate Immune Responses in Cultured Epithelial Cells
Source: PLoS Pathog. 2009 Aug 7;5(8):e1000538. doi: 10.1371/journal.ppat.1000538 (PMC2714975; doi:10.1371/journal.ppat.1000538)
Supplement: Table S2 — P values (two-tailed t test) of the fold changes listed in Table S1. (0.07 MB PDF) [file ppat.1000538.s007.pdf]

Table S2. *P* values (two-tailed *t* test) of the fold changes listed in Table S1

Probeset	Gene	ΔinvA	ΔinvA	effectorless	ΔsopB/E/E2	ΔsopB	ΔsopB	wild type	wild type	wild type
206411_s_at	ABL2	5.0E-01	5.0E-01	5.0E-01	8.5E-03	8.7E-04	1.7E-04	2.0E-05	2.0E-05	2.0E-05
231907_at	ABL2	6.0E-01	5.0E-01	3.5E-01	2.0E-05	2.0E-05	1.2E-03	2.0E-05	2.0E-05	2.0E-05
202912_at	ADM	9.8E-01	5.0E-01	3.0E-05	2.3E-05	3.0E-05	2.0E-05	2.0E-05	2.3E-05	2.0E-05
206170_at	ADRB2	5.0E-01	5.0E-01	1.2E-01	5.0E-01	4.4E-04	2.8E-03	8.8E-05	8.7E-04	6.2E-04
212543_at	AIM1	4.4E-02	1.8E-02	5.0E-01	5.1E-02	2.0E-05	3.0E-05	1.2E-03	2.0E-05	4.6E-05
210517_s_at	AKAP12	9.9E-01	5.0E-01	2.7E-05	8.8E-05	5.2E-05	2.0E-05	2.0E-05	2.0E-05	2.0E-05
227529_s_at	AKAP12	9.6E-01	7.6E-01	1.5E-01	5.0E-01	2.7E-02	3.0E-05	4.6E-05	2.0E-05	2.7E-05
227530_at	AKAP12	5.0E-01	9.3E-01	3.8E-02	5.0E-01	2.0E-05	2.3E-05	2.0E-05	2.0E-05	2.0E-05
227337_at	ANKRD37	9.0E-01	5.0E-01	5.0E-01	8.9E-02	2.1E-04	4.4E-04	1.5E-04	5.2E-05	3.9E-04
219496_at	ANKRD57	9.8E-01	9.3E-01	3.5E-04	3.5E-05	2.7E-05	2.0E-05	2.0E-05	2.0E-05	2.0E-05
227034_at	ANKRD57	9.2E-01	5.0E-01	2.7E-01	2.0E-05	2.0E-05	2.0E-05	2.0E-05	2.0E-05	2.0E-05
205239_at	AREG	1.0E+00	7.5E-01	1.9E-04	2.0E-05	2.0E-05	2.0E-05	2.0E-05	2.0E-05	2.0E-05
220382_s_at	ARHGAP28	5.0E-01	3.0E-01	9.8E-01	5.8E-01	9.0E-01	8.2E-01	1.0E+00	1.0E+00	1.0E+00
242727_at	ARL5B	1.4E-01	5.0E-01	5.0E-01	7.8E-03	2.0E-05	8.8E-05	1.9E-04	3.0E-05	7.7E-04
224797_at	ARRDC3	8.6E-01	9.9E-01	5.0E-01	1.9E-04	2.0E-05	2.0E-05	2.0E-05	2.0E-05	2.0E-05
202672_s_at	ATF3	8.4E-01	9.4E-01	5.0E-01	2.0E-05	2.0E-05	2.0E-05	2.0E-05	2.0E-05	2.0E-05
1554980_a_at	ATF3	3.0E-01	5.0E-01	9.3E-01	4.4E-04	7.7E-04	1.5E-04	2.0E-05	2.7E-05	1.0E-04
225557_at	AXUD1	5.0E-01	7.6E-01	5.0E-01	7.8E-05	2.0E-05	5.2E-05	2.0E-05	6.8E-05	2.7E-05
225612_s_at	B3GNT5	5.0E-01	5.0E-01	1.5E-01	2.0E-05	2.0E-05	2.0E-05	2.0E-05	2.0E-05	2.0E-05
1554835_a_at	B3GNT5	2.1E-01	2.3E-01	5.0E-01	2.7E-02	2.1E-04	4.9E-04	1.9E-04	7.7E-04	2.7E-04
221485_at	B4GALT5	5.0E-01	5.0E-01	5.0E-01	2.0E-03	2.0E-05	2.0E-05	2.0E-05	2.0E-05	2.0E-05
204032_at	BCAR3	5.0E-01	5.0E-01	2.4E-01	3.9E-04	7.8E-05	2.0E-05	2.0E-05	2.0E-05	2.0E-05
1557257_at	BCL10	5.0E-01	1.5E-01	1.2E-02	1.5E-03	1.7E-04	1.3E-04	4.6E-05	1.3E-04	3.1E-04
204908_s_at	BCL3	1.5E-01	5.1E-02	5.0E-01	4.9E-04	2.7E-05	8.8E-05	2.0E-05	7.8E-05	1.7E-04
201169_s_at	BHLHB2	5.0E-01	7.5E-01	5.0E-01	2.5E-01	2.0E-05	2.0E-05	2.0E-05	2.0E-05	2.0E-05
201170_s_at	BHLHB2	1.0E+00	1.0E+00	8.4E-01	1.9E-04	2.0E-05	2.0E-05	2.0E-05	2.0E-05	2.0E-05
210538_s_at	BIRC3	3.1E-01	1.2E-03	3.1E-04	2.0E-02	7.8E-05	5.2E-05	2.7E-05	2.0E-05	3.5E-05
220178_at	C19ORF28	7.3E-01	5.0E-01	3.0E-05	2.0E-05	2.0E-05	2.3E-05	2.0E-05	2.0E-05	2.0E-05
1558834_s_at	C1ORF62	3.9E-01	3.2E-01	5.0E-01	6.7E-01	1.7E-04	7.1E-03	2.3E-05	2.0E-05	4.6E-05
219474_at	C3ORF52	5.0E-01	6.2E-01	1.3E-01	4.9E-03	6.9E-04	2.8E-03	2.3E-05	4.9E-04	4.9E-04
1553868_a_at	C5ORF36	5.0E-01	9.9E-01	9.7E-01	1.0E+00	1.0E+00	1.0E+00	1.0E+00	1.0E+00	1.0E+00
218541_s_at	C8ORF4	9.7E-01	1.2E-01	5.0E-01	1.6E-01	6.5E-03	2.0E-02	1.8E-03	1.7E-04	3.9E-04
213596_at	CASP4	5.0E-01	5.0E-01	5.0E-01	3.8E-02	2.7E-04	1.1E-04	4.0E-05	2.0E-05	2.0E-05
216598_s_at	CCL2	5.0E-01	5.0E-01	5.0E-01	4.5E-03	2.0E-05	2.0E-05	2.0E-05	2.0E-05	2.0E-05
205476_at	CCL20	5.0E-01	1.5E-02	6.5E-01	5.8E-01	2.3E-03	1.2E-02	2.1E-04	5.5E-04	4.0E-05
202769_at	CCNG2	7.9E-02	8.1E-01	1.0E+00	5.0E-01	1.0E+00	1.0E+00	1.0E+00	1.0E+00	1.0E+00
220046_s_at	CCNL1	9.4E-01	5.0E-01	5.0E-01	2.1E-02	2.0E-05	2.0E-05	2.0E-05	2.0E-05	2.0E-05
1555411_a_at	CCNL1	5.0E-01	5.0E-01	5.0E-01	1.7E-01	2.0E-05	2.0E-05	2.0E-05	2.0E-05	2.0E-05
1555827_at	CCNL1	5.0E-01	5.0E-01	5.0E-01	2.3E-01	1.8E-03	1.2E-01	3.5E-05	3.5E-04	5.5E-04
204695_at	CDC25A	5.0E-01	5.0E-01	1.0E-02	1.3E-04	8.7E-04	1.7E-04	4.0E-05	7.8E-05	4.6E-05
1555772_a_at	CDC25A	5.0E-01	5.0E-01	1.5E-01	6.0E-05	2.0E-05	3.0E-05	2.0E-05	2.0E-05	2.0E-05
204677_at	CDH5	1.7E-01	5.0E-01	9.4E-02	1.8E-02	2.7E-04	7.7E-04	2.3E-05	1.9E-04	2.3E-05
202284_s_at	CDKN1A	5.0E-01	1.0E+00	5.0E-01	4.0E-05	2.0E-05	2.0E-05	2.0E-05	2.0E-05	2.0E-05
203973_s_at	CEBPD	5.0E-01	1.7E-01	5.0E-01	2.0E-05	2.0E-05	2.0E-05	2.0E-05	2.0E-05	2.0E-05
213006_at	CEBPD	8.4E-01	9.7E-01	5.0E-01	2.4E-04	4.0E-05	8.8E-05	6.8E-05	5.2E-05	5.2E-05
204203_at	CEBPG	5.3E-01	5.0E-01	2.9E-02	2.0E-05	4.9E-04	2.0E-05	2.0E-05	2.0E-05	2.0E-05
219270_at	CHAC1	8.5E-01	5.0E-01	5.0E-01	9.6E-01	2.7E-01	2.5E-02	6.8E-05	2.3E-05	2.3E-05
228297_at	CNN3	5.0E-01	8.1E-01	5.0E-01	5.0E-01	8.8E-05	2.0E-05	2.0E-05	2.0E-05	2.0E-05
207630_s_at	CREM	5.0E-01	9.7E-01	3.2E-01	4.6E-05	2.0E-05	3.5E-04	2.0E-05	2.0E-05	2.0E-05
209967_s_at	CREM	8.9E-01	1.0E+00	1.6E-01	8.8E-05	1.9E-04	7.8E-05	4.0E-05	3.5E-05	1.7E-04
214508_x_at	CREM	5.0E-01	9.3E-01	7.0E-02	2.0E-03	1.3E-02	4.5E-03	2.5E-03	2.3E-05	1.7E-03
209101_at	CTGF	1.0E+00	1.0E+00	3.5E-04	2.7E-05	3.5E-05	2.0E-05	2.3E-05	6.8E-05	2.0E-05
209774_x_at	CXCL2	5.0E-01	5.0E-01	3.7E-03	2.3E-05	2.0E-05	2.0E-05	2.0E-05	2.0E-05	2.0E-05
1569203_at	CXCL2	8.4E-02	2.0E-02	7.5E-01	5.0E-01	3.5E-04	1.3E-02	4.9E-04	2.0E-05	6.9E-04
207850_at	CXCL3	5.0E-01	5.0E-01	5.0E-01	4.5E-03	1.9E-04	6.8E-05	4.6E-05	2.3E-05	2.3E-05
212977_at	CXCR7	5.0E-01	6.8E-01	7.5E-01	9.9E-01	1.0E+00	1.0E+00	1.0E+00	1.0E+00	1.0E+00
201289_at	CYR61	1.0E+00	1.0E+00	3.4E-03	2.7E-04	2.0E-05	2.0E-05	2.0E-05	2.0E-05	2.0E-05
210764_s_at	CYR61	9.9E-01	1.0E+00	1.5E-02	6.8E-05	2.0E-05	2.0E-05	2.0E-05	2.0E-05	2.0E-05
231919_at	DBT	5.0E-01	5.0E-01	9.9E-01	1.0E+00	1.0E+00	9.1E-01	1.0E+00	1.0E+00	1.0E+00
204602_at	DKK1	1.0E+00	1.0E+00	2.0E-05	2.0E-05	2.0E-05	2.0E-05	2.0E-05	2.0E-05	2.0E-05
1557394_at	DLGAP4	2.1E-01	2.1E-02	5.0E-01	5.0E-01	2.8E-03	2.0E-02	4.0E-05	3.0E-05	6.9E-04
231297_at	DOT1L	3.1E-01	5.0E-01	4.0E-01	5.8E-02	5.9E-03	2.0E-05	6.2E-04	1.3E-03	2.1E-04
201041_s_at	DUSP1	1.0E+00	1.0E+00	5.0E-01	2.0E-05	2.0E-05	2.7E-05	2.0E-05	2.0E-05	2.0E-05
201044_x_at	DUSP1	9.0E-01	1.0E+00	5.0E-01	7.8E-05	2.0E-05	2.3E-05	2.0E-05	2.0E-05	2.0E-05

204794_at	DUSP2	3.0E-01	6.2E-01	1.8E-02	3.0E-03	3.9E-04	5.5E-04	1.5E-04	4.9E-04	2.3E-05
204014_at	DUSP4	9.9E-01	1.0E+00	4.4E-04	4.6E-05	6.8E-05	6.8E-05	2.7E-05	1.9E-04	1.1E-04
204015_s_at	DUSP4	1.0E+00	8.6E-01	9.7E-04	3.1E-04	2.0E-05	2.0E-05	2.0E-05	2.0E-05	2.0E-05
209457_at	DUSP5	5.0E-01	5.3E-01	3.2E-01	4.4E-04	2.0E-05	2.0E-05	2.0E-05	2.0E-05	2.0E-05
208891_at	DUSP6	1.0E+00	9.9E-01	5.0E-01	5.0E-01	4.0E-05	5.2E-05	2.7E-05	2.0E-05	2.0E-05
208892_s_at	DUSP6	7.6E-01	9.4E-01	5.1E-02	4.0E-01	2.0E-05	2.0E-05	2.0E-05	2.0E-05	2.0E-05
208893_s_at	DUSP6	9.3E-01	7.7E-01	1.4E-01	5.0E-01	4.9E-04	1.5E-03	2.3E-05	2.0E-05	2.1E-04
206374_at	DUSP8	1.5E-01	5.0E-01	5.0E-01	2.4E-01	1.8E-02	1.4E-02	1.1E-04	6.9E-04	6.2E-04
201693_s_at	EGR1	5.0E-01	8.1E-01	1.1E-01	5.0E-01	2.0E-05	2.0E-05	2.0E-05	2.0E-05	2.0E-05
201694_s_at	EGR1	5.0E-01	8.9E-02	8.4E-02	6.2E-04	2.0E-05	2.0E-05	2.0E-05	2.0E-05	2.0E-05
227404_s_at	EGR1	5.0E-01	5.0E-01	1.8E-02	2.0E-05	2.0E-05	2.0E-05	2.0E-05	2.0E-05	2.0E-05
206115_at	EGR3	8.4E-02	5.0E-01	8.4E-01	9.7E-01	3.5E-05	2.3E-03	2.7E-04	1.5E-04	2.1E-04
218696_at	EIF2AK3	5.0E-01	5.0E-01	5.0E-01	2.7E-04	2.0E-05	2.0E-05	2.0E-05	2.0E-05	2.0E-05
201303_at	EIF4A3	5.0E-01	4.8E-01	1.5E-02	1.7E-04	2.0E-05	2.0E-05	2.0E-05	2.0E-05	2.0E-05
235592_at	ELL2	1.0E+00	6.2E-01	1.5E-03	5.4E-03	2.7E-05	1.7E-04	2.0E-05	2.0E-05	2.7E-05
201324_at	EMP1	9.6E-01	5.0E-01	2.2E-01	1.8E-01	5.2E-05	2.0E-05	2.0E-05	3.5E-05	2.0E-05
201325_s_at	EMP1	5.0E-01	9.7E-01	5.0E-01	2.4E-01	2.7E-04	1.5E-04	1.1E-04	2.0E-05	2.3E-05
242868_at	EPAS1	5.0E-01	5.0E-01	5.0E-01	2.8E-03	4.0E-05	2.1E-04	2.3E-05	2.0E-05	3.1E-04
203499_at	EPHA2	9.9E-01	9.2E-01	6.2E-02	4.0E-05	2.0E-05	2.0E-05	2.0E-05	2.0E-05	2.0E-05
205767_at	EREG	7.6E-01	5.0E-01	8.8E-05	2.0E-05	2.3E-05	2.0E-05	2.0E-05	2.0E-05	2.0E-05
224657_at	ERRFI1	1.0E+00	1.0E+00	5.0E-01	5.2E-05	2.0E-05	2.0E-05	2.0E-05	2.0E-05	2.0E-05
224454_at	ETNK1	5.2E-01	2.5E-03	1.4E-02	3.9E-04	2.3E-05	4.0E-05	6.8E-05	3.0E-05	1.1E-04
1555355_a_at	ETS1	7.8E-03	4.5E-03	2.8E-01	4.4E-02	3.0E-03	3.7E-03	1.5E-04	3.5E-04	1.9E-04
201328_at	ETS2	5.0E-01	1.0E+00	1.0E+00	5.0E-01	1.7E-04	2.0E-05	2.3E-05	2.7E-05	2.0E-05
201329_s_at	ETS2	8.6E-01	1.0E+00	1.0E+00	8.8E-05	2.0E-05	2.0E-05	2.0E-05	2.0E-05	2.0E-05
213506_at	F2RL1	8.0E-01	9.3E-01	5.0E-01	5.0E-01	2.7E-05	2.0E-05	2.0E-05	2.0E-05	3.5E-05
204363_at	F3	1.0E+00	5.0E-01	4.0E-05	2.7E-05	6.0E-05	6.8E-05	2.3E-05	4.0E-05	6.0E-05
227410_at	FAM43A	7.9E-01	9.9E-01	5.0E-01	5.0E-01	1.0E+00	1.0E+00	1.0E+00	1.0E+00	1.0E+00
232202_at	FAM83B	5.0E-01	9.5E-01	5.0E-01	4.3E-01	2.0E-05	1.5E-04	2.0E-05	2.0E-05	1.0E-04
223240_at	FBXO8	5.0E-01	1.0E+00	9.4E-01	5.0E-01	1.0E+00	9.8E-01	1.0E+00	1.0E+00	1.0E+00
205650_s_at	FGA	5.5E-01	5.0E-01	5.0E-01	5.0E-01	2.0E-05	2.4E-04	2.7E-04	2.0E-05	2.7E-04
204988_at	FGB	2.7E-01	5.0E-01	3.0E-01	5.0E-01	3.1E-04	3.1E-04	2.7E-04	7.7E-04	7.7E-04
204421_s_at	FGF2	5.0E-01	5.0E-01	5.0E-01	5.0E-01	1.7E-04	3.1E-04	8.8E-05	1.0E-04	1.3E-03
226621_at	FGG	5.0E-01	6.6E-01	5.0E-01	2.7E-05	2.0E-05	2.0E-05	3.0E-05	2.0E-05	2.0E-05
243309_at	FLJ27352	5.0E-01	6.8E-01	3.8E-01	3.5E-05	2.0E-05	2.3E-05	2.0E-05	2.0E-05	2.3E-05
229521_at	FLJ36031	4.7E-02	5.0E-01	5.0E-01	3.2E-01	8.7E-04	4.9E-04	2.0E-05	2.7E-05	8.8E-05
239331_at	FLJ43663	9.9E-01	7.9E-02	5.0E-01	2.7E-02	2.3E-05	3.0E-05	2.0E-05	2.0E-05	2.0E-05
219250_s_at	FLRT3	5.0E-01	9.1E-01	5.0E-01	3.1E-02	2.0E-02	1.9E-04	4.0E-05	2.3E-05	2.3E-05
222853_at	FLRT3	8.0E-01	9.1E-01	3.8E-01	5.5E-04	1.3E-04	9.7E-04	2.7E-05	1.0E-04	2.3E-05
1559060_a_at	FNIP1	9.9E-01	2.0E-02	6.5E-01	5.0E-01	8.7E-04	2.1E-04	4.6E-05	5.2E-05	4.9E-04
209189_at	FOS	9.9E-01	9.9E-01	5.0E-01	2.0E-05	2.0E-05	2.0E-05	2.0E-05	2.0E-05	2.0E-05
202768_at	FOSB	2.6E-01	9.1E-01	5.5E-01	2.0E-02	4.5E-03	1.5E-03	3.5E-05	6.2E-04	1.5E-03
204420_at	FOSL1	7.2E-01	5.0E-01	3.0E-03	1.3E-04	2.0E-05	2.0E-05	2.0E-05	2.0E-05	2.0E-05
218880_at	FOSL2	5.0E-01	1.8E-01	4.4E-02	2.0E-05	2.0E-05	2.0E-05	2.0E-05	2.0E-05	2.0E-05
225262_at	FOSL2	5.0E-01	5.0E-01	4.1E-02	2.0E-05	2.3E-05	2.0E-05	2.0E-05	2.0E-05	2.7E-05
227475_at	FOXQ1	5.0E-01	5.0E-01	2.9E-02	2.0E-05	2.0E-05	2.0E-05	2.0E-05	2.0E-05	2.0E-05
213056_at	FRMD4B	5.0E-01	4.7E-01	5.0E-01	3.6E-02	7.8E-05	1.0E-04	2.0E-05	2.0E-05	7.8E-05
210220_at	FZD2	2.1E-04	5.0E-01	1.0E+00	1.0E+00	1.0E+00	1.0E+00	1.0E+00	1.0E+00	1.0E+00
207574_s_at	GADD45B	8.7E-01	9.7E-01	5.0E-01	5.0E-01	2.0E-05	4.9E-04	2.0E-05	2.0E-05	2.0E-05
209304_x_at	GADD45B	9.3E-01	8.9E-01	5.0E-01	5.0E-01	2.0E-03	2.0E-05	2.0E-05	2.7E-05	6.8E-05
209305_s_at	GADD45B	5.3E-01	5.0E-01	5.0E-01	5.6E-01	6.0E-05	1.1E-03	2.0E-05	2.3E-05	8.8E-05
213560_at	GADD45B	6.0E-01	5.0E-01	4.9E-01	5.0E-01	4.8E-01	1.8E-02	2.7E-04	1.7E-03	3.1E-04
204457_s_at	GAS1	5.0E-01	1.0E+00	1.0E+00	1.0E+00	1.0E+00	1.0E+00	1.0E+00	1.0E+00	1.0E+00
219508_at	GCNT3	3.9E-01	5.0E-01	2.7E-01	5.0E-01	3.9E-04	2.3E-05	1.5E-04	3.5E-05	3.5E-05
221577_x_at	GDF15	5.0E-01	7.7E-01	5.0E-01	2.0E-05	2.0E-05	2.0E-05	2.0E-05	2.0E-05	2.0E-05
204472_at	GEM	5.0E-01	5.6E-01	4.3E-01	3.1E-04	2.0E-05	2.0E-05	2.0E-05	2.0E-05	2.0E-05
205100_at	GFPT2	5.9E-03	2.4E-04	2.1E-01	5.0E-01	9.7E-04	2.1E-04	2.0E-05	2.0E-05	3.5E-05
204222_s_at	GLIPR1	5.0E-01	5.0E-01	1.7E-02	2.0E-02	5.4E-03	7.1E-03	2.0E-05	6.0E-05	6.8E-05
226136_at	GLIPR1	5.0E-01	6.2E-01	5.0E-01	5.0E-01	3.6E-02	7.1E-03	2.0E-05	1.7E-04	2.1E-04
226142_at	GLIPR1	7.5E-01	7.4E-01	3.8E-02	2.7E-02	4.1E-02	2.8E-03	2.3E-05	2.0E-05	2.0E-05
223079_s_at	GLS	5.0E-01	5.0E-01	6.9E-04	2.0E-05	2.0E-05	2.0E-05	2.0E-05	2.0E-05	2.0E-05
219936_s_at	GPR87	5.0E-01	7.7E-01	1.9E-04	2.0E-05	2.0E-05	2.0E-05	2.0E-05	2.0E-05	2.0E-05
203108_at	GPRC5A	5.0E-01	9.8E-01	2.0E-05	2.0E-05	2.0E-05	2.0E-05	2.0E-05	2.0E-05	2.0E-05
232290_at	GPRC5A	7.6E-01	6.9E-01	5.8E-02	3.5E-04	5.2E-05	5.4E-02	2.3E-05	1.5E-04	1.8E-03
218706_s_at	GRAMD3	5.0E-01	5.0E-01	5.0E-01	2.4E-01	2.0E-05	2.0E-05	2.0E-05	2.0E-05	2.0E-05
212434_at	GRPEL1	5.0E-01	5.0E-01	3.7E-03	5.2E-05	2.0E-05	4.6E-05	2.0E-05	2.0E-05	2.0E-05
223541_at	HAS3	1.5E-01	5.0E-01	8.7E-04	7.4E-02	1.8E-01	1.2E-02	6.8E-05	9.7E-04	9.7E-04

1557169_x_at	HCG11	5.0E-01	5.0E-01	4.3E-01	1.8E-03	1.0E+00	1.0E+00	1.0E+00	1.0E+00	1.0E+00
232026_at	HERC4	6.8E-01	7.4E-01	3.1E-02	5.1E-02	1.1E-04	6.8E-05	5.5E-04	1.3E-04	1.7E-04
203394_s_at	HES1	6.5E-01	5.0E-01	3.1E-01	1.4E-02	3.0E-05	4.6E-05	2.0E-05	4.6E-05	2.1E-04
218839_at	HEY1	3.3E-01	5.0E-01	1.0E+00	1.0E+00	1.0E+00	1.0E+00	1.0E+00	1.0E+00	1.0E+00
202934_at	HK2	9.4E-01	5.0E-01	5.8E-02	1.3E-04	2.1E-04	3.0E-05	5.2E-05	3.0E-05	1.3E-04
205579_at	HRH1	3.1E-01	5.0E-01	6.5E-03	3.4E-03	2.0E-05	3.0E-05	3.0E-05	2.0E-05	2.0E-05
221667_s_at	HSPB8	3.7E-01	7.7E-01	5.0E-01	5.0E-01	2.3E-02	4.6E-05	2.0E-05	8.8E-05	7.8E-05
208937_s_at	ID1	8.7E-01	8.7E-01	5.0E-01	2.0E-05	2.0E-05	2.0E-05	2.0E-05	2.0E-05	2.0E-05
201631_s_at	IER3	1.0E+00	2.5E-02	2.1E-04	2.0E-05	2.0E-05	2.0E-05	2.0E-05	2.0E-05	2.0E-05
218611_at	IER5	5.0E-01	5.0E-01	2.2E-01	4.0E-05	2.0E-05	2.0E-05	2.0E-05	2.0E-05	2.0E-05
206332_s_at	IFI16	5.0E-01	5.0E-01	5.0E-01	4.9E-03	2.0E-05	2.0E-05	2.0E-05	2.0E-05	2.0E-05
208966_x_at	IFI16	5.0E-01	5.0E-01	5.0E-01	8.8E-05	2.0E-05	2.0E-05	2.0E-05	2.0E-05	2.0E-05
205302_at	IGFBP1	2.5E-01	4.9E-01	5.0E-01	6.0E-05	5.2E-05	2.0E-05	2.0E-05	3.5E-05	5.2E-05
206924_at	IL11	9.3E-01	7.3E-01	8.0E-01	1.3E-01	1.5E-04	1.1E-04	8.8E-05	6.8E-05	1.7E-04
210118_s_at	IL1A	5.0E-01	8.3E-01	5.0E-01	1.8E-02	4.1E-02	1.0E-04	2.0E-05	5.2E-05	2.0E-05
202948_at	IL1R1	7.0E-01	8.9E-01	5.0E-01	1.4E-02	2.0E-05	1.9E-04	2.3E-05	4.0E-05	2.0E-05
203233_at	IL4R	5.0E-01	5.0E-01	7.8E-05	4.9E-03	4.1E-03	5.5E-04	4.0E-05	1.3E-04	4.6E-05
202859_x_at	IL8	3.0E-03	3.8E-01	6.9E-01	5.4E-02	2.7E-05	2.0E-05	2.0E-05	3.5E-05	2.0E-05
211506_s_at	IL8	1.9E-01	5.0E-01	5.3E-01	5.0E-01	3.5E-05	2.0E-05	2.7E-05	2.0E-05	2.0E-05
1557174_a_at	IRAK1BP1	5.0E-01	7.9E-01	9.9E-01	1.0E+00	1.0E+00	1.0E+00	1.0E+00	1.0E+00	1.0E+00
202531_at	IRF1	5.0E-01	5.0E-01	5.0E-01	3.1E-01	1.7E-04	1.3E-04	2.0E-05	2.3E-05	1.0E-04
204698_at	ISG20	5.0E-01	5.0E-01	5.0E-01	2.6E-01	4.9E-03	7.8E-03	1.5E-04	2.0E-05	2.5E-03
219361_s_at	ISG20L1	5.0E-01	5.0E-01	4.3E-01	1.7E-04	2.3E-05	3.5E-05	1.7E-04	6.0E-05	1.5E-04
205032_at	ITGA2	5.0E-01	5.0E-01	4.8E-01	5.0E-01	3.4E-01	1.1E-04	1.5E-04	1.1E-04	8.8E-05
227314_at	ITGA2	6.3E-01	1.0E+00	7.0E-01	8.9E-01	5.2E-05	2.7E-05	2.0E-05	2.0E-05	2.0E-05
213146_at	JMJD3	6.9E-01	3.3E-01	1.4E-01	9.3E-03	3.0E-03	3.5E-04	6.0E-05	1.3E-04	2.1E-04
212723_at	JMJD6	5.0E-01	5.0E-01	8.7E-04	2.3E-05	2.0E-05	2.0E-05	2.0E-05	2.0E-05	2.0E-05
226352_at	JMY	5.0E-01	5.0E-01	5.0E-01	3.5E-01	1.0E+00	1.0E+00	1.0E+00	1.0E+00	1.0E+00
243446_at	JUB	3.8E-01	3.4E-01	6.3E-01	5.0E-01	4.7E-02	9.4E-02	1.8E-03	2.3E-03	5.5E-04
201464_x_at	JUN	6.7E-01	5.0E-01	2.8E-03	7.8E-05	1.7E-04	3.5E-04	5.2E-05	7.8E-05	1.1E-04
201465_s_at	JUN	9.2E-01	8.5E-01	2.6E-01	3.1E-04	5.9E-03	2.0E-02	2.0E-05	8.7E-04	2.1E-04
201466_s_at	JUN	1.3E-01	5.0E-01	5.0E-01	3.0E-05	1.1E-04	3.5E-05	2.0E-05	2.0E-05	5.2E-05
213281_at	JUN	8.5E-01	5.0E-01	1.9E-01	8.7E-04	6.2E-04	5.1E-02	5.2E-05	6.0E-05	5.2E-05
201473_at	JUNB	2.4E-01	5.0E-01	1.5E-03	2.0E-05	2.0E-05	2.0E-05	2.0E-05	2.0E-05	2.0E-05
228325_at	KIAA0146	6.8E-01	5.0E-01	5.0E-01	2.3E-05	2.3E-05	2.3E-05	2.0E-05	2.0E-05	2.0E-05
202386_s_at	KIAA0430	5.0E-01	5.0E-01	5.0E-01	5.8E-01	1.0E+00	1.0E+00	1.0E+00	1.0E+00	1.0E+00
225582_at	KIAA1754	9.9E-01	8.4E-01	1.1E-01	6.0E-05	2.0E-05	2.0E-05	2.0E-05	2.0E-05	2.0E-05
225193_at	KIAA1967	9.9E-01	5.0E-01	5.8E-01	1.0E+00	1.0E+00	1.0E+00	1.0E+00	1.0E+00	1.0E+00
219371_s_at	KLF2	5.0E-01	6.3E-01	1.0E+00	9.5E-01	3.7E-01	2.9E-02	4.6E-05	7.7E-04	1.0E-04
209212_s_at	KLF5	5.0E-01	5.0E-01	5.0E-01	2.0E-05	2.0E-05	2.0E-05	2.0E-05	2.0E-05	2.0E-05
208960_s_at	KLF6	6.2E-01	9.3E-01	5.0E-01	2.0E-02	7.7E-04	1.1E-03	2.0E-05	2.0E-05	1.0E-04
208961_s_at	KLF6	5.0E-01	5.0E-01	5.0E-01	1.9E-04	2.0E-05	6.8E-05	2.0E-05	2.0E-05	2.0E-05
224606_at	KLF6	7.7E-01	1.0E+00	5.0E-01	4.6E-05	2.0E-05	2.0E-05	2.0E-05	2.0E-05	2.0E-05
1555832_s_at	KLF6	8.7E-01	9.8E-01	5.0E-01	1.1E-02	2.0E-05	2.0E-05	2.0E-05	2.0E-05	2.0E-05
226158_at	KLHL24	5.0E-01	5.0E-01	5.0E-01	2.5E-03	1.0E+00	1.0E+00	1.0E+00	1.0E+00	1.0E+00
205157_s_at	KRT17	5.0E-01	5.0E-01	2.0E-05	2.0E-05	2.0E-05	2.0E-05	2.0E-05	2.0E-05	2.0E-05
212236_x_at	KRT17	5.0E-01	5.0E-01	2.0E-05	2.0E-05	2.0E-05	2.0E-05	2.0E-05	2.0E-05	2.0E-05
202067_s_at	LDLR	5.3E-01	9.9E-01	8.4E-01	5.0E-01	1.9E-04	2.0E-03	2.0E-05	6.0E-05	1.3E-04
202068_s_at	LDLR	1.0E+00	1.0E+00	9.8E-01	5.0E-01	2.0E-05	2.0E-05	2.0E-05	2.0E-05	2.0E-05
217173_s_at	LDLR	7.9E-01	1.0E+00	5.0E-01	5.0E-01	3.7E-03	2.1E-02	2.0E-05	2.0E-05	2.0E-05
207170_s_at	LETMD1	5.0E-01	5.0E-01	7.6E-01	9.2E-01	1.0E+00	1.0E+00	1.0E+00	1.0E+00	1.0E+00
242424_at	LETMD1	9.2E-01	5.0E-01	5.0E-01	1.0E+00	1.0E+00	9.9E-01	1.0E+00	1.0E+00	1.0E+00
228762_at	LFNG	5.0E-01	1.1E-01	5.0E-01	9.9E-01	1.0E+00	9.9E-01	1.0E+00	1.0E+00	1.0E+00
214045_at	LIAS	6.7E-01	5.0E-01	5.0E-01	9.9E-01	1.0E+00	1.0E+00	1.0E+00	1.0E+00	1.0E+00
205266_at	LIF	6.7E-01	5.0E-01	2.3E-01	5.0E-01	1.1E-01	1.7E-01	1.7E-04	2.3E-03	3.9E-04
241140_at	LMO7	9.8E-01	3.8E-02	5.0E-01	5.0E-01	8.8E-05	1.5E-02	2.0E-05	1.0E-04	6.9E-04
1556377_s_at	LMO7	6.0E-01	2.3E-01	5.0E-01	4.4E-01	1.0E-04	3.1E-04	5.5E-04	1.5E-04	1.7E-04
231828_at	LOC253039	5.0E-01	5.0E-01	7.3E-01	1.0E+00	1.0E+00	1.0E+00	1.0E+00	1.0E+00	1.0E+00
236076_at	LOC257396	5.0E-01	7.9E-01	9.5E-01	1.0E+00	1.0E+00	1.0E+00	1.0E+00	1.0E+00	1.0E+00
235151_at	LOC283357	6.2E-01	5.0E-01	1.0E+00	9.6E-01	1.0E+00	1.0E+00	1.0E+00	1.0E+00	1.0E+00
1556064_at	LOC284926	5.0E-01	1.0E+00	1.0E+00	1.0E+00	1.0E+00	1.0E+00	1.0E+00	1.0E+00	1.0E+00
222662_at	LOC286044	4.0E-01	5.0E-01	5.0E-01	2.5E-01	6.2E-04	3.7E-03	2.0E-05	2.0E-05	2.0E-05
227940_at	LOC339803	5.0E-01	1.0E+00	9.7E-01	1.0E+00	1.0E+00	1.0E+00	1.0E+00	1.0E+00	1.0E+00
243009_at	LOC441242	9.9E-01	8.8E-01	8.4E-01	1.0E+00	1.0E+00	1.0E+00	1.0E+00	1.0E+00	1.0E+00
226977_at	LOC492311	5.0E-01	5.0E-01	1.0E+00	1.0E+00	1.0E+00	1.0E+00	1.0E+00	1.0E+00	1.0E+00
241874_at	LOC492311	6.1E-01	5.0E-01	5.0E-01	8.1E-01	1.0E+00	1.0E+00	1.0E+00	1.0E+00	1.0E+00
220770_s_at	LOC63920	5.0E-01	5.0E-01	5.0E-01	1.0E+00	1.0E+00	1.0E+00	1.0E+00	1.0E+00	1.0E+00

242396_at	LOC644192	9.7E-01	7.6E-01	5.0E-01	9.6E-01	1.0E+00	1.0E+00	1.0E+00	1.0E+00	1.0E+00
1555978_s_at	LOC727918	5.0E-01	5.0E-01	5.0E-01	5.0E-01	1.9E-04	4.4E-04	1.5E-04	2.0E-05	4.0E-05
217122_s_at	LOC728661	5.0E-01	5.0E-01	5.0E-01	9.9E-01	1.0E+00	1.0E+00	1.0E+00	1.0E+00	1.0E+00
239343_at	LOC728705	5.0E-01	5.0E-01	6.2E-01	1.0E+00	1.0E+00	1.0E+00	1.0E+00	1.0E+00	1.0E+00
228536_at	LOC90826	5.6E-01	5.0E-01	7.8E-03	2.4E-04	4.0E-05	3.5E-05	2.3E-05	3.0E-05	2.3E-05
223533_at	LRRC8C	5.0E-01	5.0E-01	1.4E-01	1.8E-01	4.9E-04	2.3E-05	2.0E-05	2.0E-05	2.0E-05
218559_s_at	MAFB	8.8E-01	2.9E-02	9.9E-01	9.2E-01	2.5E-02	1.2E-02	2.0E-05	2.0E-05	2.1E-04
205193_at	MAFF	5.0E-01	5.0E-01	2.4E-04	2.0E-05	2.3E-05	5.2E-05	2.0E-05	2.3E-05	2.0E-05
36711_at	MAFF	5.7E-01	1.8E-02	0.0E+00	0.0E+00	0.0E+00	0.0E+00	0.0E+00	0.0E+00	0.0E+00
206750_at	MAFK	4.8E-01	5.0E-01	4.8E-01	1.3E-04	5.5E-04	3.5E-04	3.0E-05	4.6E-05	1.3E-04
226206_at	MAFK	9.7E-01	9.4E-01	4.4E-04	2.0E-05	2.0E-05	2.0E-05	2.0E-05	2.0E-05	2.3E-05
224480_s_at	MAG1	7.7E-01	9.3E-01	6.8E-01	5.0E-01	2.0E-05	3.5E-04	1.1E-04	2.0E-05	1.5E-04
205027_s_at	MAP3K8	5.0E-01	6.0E-01	4.9E-01	2.0E-05	4.0E-05	2.0E-05	2.0E-05	2.0E-05	3.0E-05
235421_at	MAP3K8	5.3E-01	3.3E-02	1.8E-01	4.7E-01	4.9E-04	1.8E-02	2.0E-05	1.7E-04	3.9E-04
236067_at	MBNL2	5.0E-01	5.0E-01	2.7E-01	3.4E-01	4.9E-04	1.1E-04	4.6E-05	1.3E-04	2.4E-04
236699_at	MBNL2	1.0E+00	5.0E-01	5.0E-01	2.1E-02	2.0E-05	6.2E-04	2.0E-05	2.0E-05	2.0E-05
200796_s_at	MCL1	5.0E-01	5.0E-01	8.5E-03	3.0E-05	2.3E-05	2.0E-05	2.0E-05	2.3E-05	3.5E-05
200797_s_at	MCL1	5.0E-01	5.0E-01	1.2E-03	1.0E-04	2.0E-05	2.0E-05	2.0E-05	2.0E-05	2.0E-05
200798_x_at	MCL1	5.0E-01	5.0E-01	6.0E-05	2.0E-05	2.0E-05	2.0E-05	2.0E-05	2.0E-05	2.0E-05
214056_at	MCL1	9.9E-01	5.0E-01	5.0E-01	2.7E-04	2.0E-05	3.0E-05	3.0E-05	2.7E-05	2.0E-05
214057_at	MCL1	5.7E-01	5.0E-01	5.0E-01	3.3E-02	5.2E-05	2.0E-05	1.7E-04	2.0E-05	2.0E-05
227175_at	MCL1	5.2E-01	5.0E-01	5.0E-01	1.5E-01	4.4E-04	1.8E-02	3.9E-04	4.6E-05	9.7E-04
212830_at	MEGF9	9.8E-01	1.0E+00	9.5E-01	5.0E-01	1.0E+00	1.0E+00	1.0E+00	1.0E+00	1.0E+00
214696_at	MGC14376	8.0E-01	1.0E+00	5.4E-02	2.1E-04	8.8E-05	5.2E-05	2.0E-05	2.0E-05	6.0E-05
221477_s_at	MGC5618	2.3E-05	2.0E-05	4.0E-05	3.5E-05	2.0E-05	2.0E-05	3.5E-05	2.0E-05	2.0E-05
239549_at	M-RIP	9.7E-01	5.0E-01	7.4E-02	2.4E-01	1.2E-03	1.0E-01	4.6E-05	2.7E-04	1.9E-04
1570588_at	M-RIP	8.5E-01	4.4E-01	5.0E-01	4.7E-02	1.3E-03	1.2E-01	6.8E-05	2.7E-04	1.5E-03
217165_x_at	MT1F	5.0E-01	3.7E-01	1.3E-01	3.4E-01	7.7E-04	2.3E-03	1.1E-04	3.9E-04	2.0E-05
211456_x_at	MT1P2	5.0E-01	5.0E-01	1.0E-02	5.0E-01	2.7E-04	1.1E-04	1.3E-04	2.4E-04	3.5E-05
204326_x_at	MT1X	5.0E-01	5.0E-01	1.0E-01	3.7E-01	2.3E-02	6.8E-05	2.0E-05	2.3E-03	3.0E-05
208581_x_at	MT1X	5.0E-01	5.0E-01	4.4E-04	2.2E-01	3.5E-04	1.3E-04	6.8E-05	8.8E-05	6.0E-05
212185_x_at	MT2A	5.0E-01	1.1E-01	1.1E-03	5.0E-01	2.0E-05	2.0E-05	2.0E-05	2.0E-05	2.0E-05
225344_at	NCOA7	4.5E-01	5.0E-01	5.0E-01	2.0E-05	2.0E-05	2.0E-05	2.0E-05	2.0E-05	2.0E-05
203574_at	NFIL3	5.0E-01	1.0E+00	1.0E+00	2.0E-05	2.0E-05	2.0E-05	2.0E-05	2.0E-05	2.0E-05
201502_s_at	NFKBIA	9.3E-03	5.0E-01	1.7E-01	2.0E-05	5.9E-03	2.0E-05	2.0E-05	2.0E-05	2.0E-05
223217_s_at	NFKBIZ	5.0E-01	5.0E-01	1.8E-02	2.3E-05	2.0E-05	2.0E-05	2.0E-05	2.0E-05	2.0E-05
223218_s_at	NFKBIZ	9.6E-01	5.0E-01	7.1E-03	2.0E-05	2.0E-05	2.0E-05	2.0E-05	2.0E-05	2.0E-05
202585_s_at	NFX1	5.0E-01	8.1E-01	8.9E-01	9.9E-01	9.9E-01	1.0E+00	1.0E+00	1.0E+00	1.0E+00
202237_at	NNMT	5.0E-01	5.0E-01	5.0E-01	1.9E-04	2.0E-05	2.0E-05	2.3E-05	2.0E-05	2.0E-05
202238_s_at	NNMT	5.0E-01	5.0E-01	5.0E-01	6.0E-05	2.0E-05	2.0E-05	2.0E-05	2.0E-05	2.0E-05
231798_at	NOG	5.0E-01	9.3E-01	9.3E-01	9.3E-01	1.0E+00	1.0E+00	1.0E+00	1.0E+00	1.0E+00
244855_at	NQO2	5.0E-01	8.5E-01	4.3E-01	4.8E-01	1.5E-01	2.3E-03	8.7E-04	9.7E-04	2.5E-03
202340_x_at	NR4A1	5.0E-01	5.0E-01	1.5E-01	2.4E-04	2.0E-05	2.0E-05	2.0E-05	4.6E-05	2.0E-05
204621_s_at	NR4A2	1.0E+00	1.0E+00	5.0E-01	7.8E-05	1.0E-04	8.8E-05	2.0E-05	1.3E-04	7.8E-05
235739_at	NR4A2	3.5E-01	5.0E-01	5.0E-01	1.6E-01	4.0E-05	2.0E-05	2.0E-05	5.2E-05	2.0E-05
207978_s_at	NR4A3	8.8E-01	1.0E+00	6.2E-01	1.5E-04	2.0E-05	2.0E-05	2.0E-05	2.0E-05	2.3E-05
209959_at	NR4A3	9.9E-01	6.7E-01	5.0E-01	4.9E-04	4.0E-05	2.1E-04	2.3E-05	2.3E-05	6.8E-05
204435_at	NUPL1	1.0E+00	4.5E-01	1.1E-04	2.3E-05	2.0E-05	2.3E-05	2.0E-05	2.0E-05	2.0E-05
219334_s_at	OBFC2A	8.1E-01	7.5E-01	5.0E-01	5.0E-01	4.9E-03	2.7E-04	1.1E-04	2.4E-04	2.4E-04
222872_x_at	OBFC2A	5.0E-01	7.3E-01	5.0E-01	2.1E-04	2.3E-05	2.0E-05	2.0E-05	2.0E-05	2.0E-05
233085_s_at	OBFC2A	9.9E-01	5.0E-01	5.0E-01	7.8E-03	2.0E-05	2.0E-05	2.0E-05	2.0E-05	2.0E-05
205729_at	OSMR	5.0E-01	6.0E-01	5.0E-01	2.7E-05	4.0E-05	2.0E-05	6.0E-05	6.8E-05	2.0E-05
226140_s_at	OTUD1	2.4E-01	5.0E-01	5.0E-01	2.0E-05	1.9E-04	3.1E-04	2.0E-05	2.0E-05	2.0E-05
243296_at	PBEF1	9.9E-01	3.3E-01	7.5E-01	5.0E-01	2.0E-05	1.1E-04	2.0E-05	2.0E-05	2.0E-05
205534_at	PCDH7	5.0E-01	2.2E-01	5.0E-01	5.0E-01	2.0E-05	3.5E-05	2.0E-05	2.3E-05	2.0E-05
205535_s_at	PCDH7	5.0E-01	1.6E-01	5.6E-01	5.0E-01	1.7E-02	4.5E-03	3.5E-04	2.3E-05	2.0E-05
232382_s_at	PCMTD1	5.0E-01	5.0E-01	5.0E-01	5.0E-01	1.0E+00	9.8E-01	1.0E+00	1.0E+00	1.0E+00
238902_at	PCMTD1	8.4E-01	5.0E-01	5.0E-01	7.8E-01	1.0E+00	1.0E+00	1.0E+00	1.0E+00	1.0E+00
230109_at	PDE7B	7.2E-01	1.0E+00	9.7E-01	9.9E-01	1.0E+00	1.0E+00	1.0E+00	1.0E+00	1.0E+00
202464_s_at	PFKFB3	5.0E-01	5.0E-01	5.0E-01	2.1E-01	4.0E-01	4.0E-05	2.0E-05	2.7E-05	5.2E-05
217996_at	PHLDA1	1.0E+00	5.0E-01	2.0E-05	4.0E-05	2.0E-05	2.0E-05	2.0E-05	2.0E-05	2.0E-05
217997_at	PHLDA1	1.0E+00	8.0E-01	2.0E-05	3.1E-02	2.0E-05	2.0E-05	2.0E-05	2.0E-05	2.0E-05
218000_s_at	PHLDA1	9.6E-01	1.6E-01	1.1E-04	2.0E-05	2.0E-05	2.0E-05	2.0E-05	2.0E-05	2.0E-05
225842_at	PHLDA1	5.0E-01	9.0E-01	6.2E-02	4.4E-02	5.5E-04	2.0E-05	2.3E-05	2.0E-05	3.0E-05
239102_s_at	PICALM	9.7E-01	2.0E-01	5.5E-04	2.0E-03	6.9E-04	7.9E-02	4.0E-05	8.8E-05	2.4E-04
210845_s_at	PLAUR	8.8E-01	6.3E-01	5.0E-01	6.5E-03	2.1E-04	2.3E-05	2.0E-05	2.0E-05	2.0E-05
211924_s_at	PLAUR	5.0E-01	5.0E-01	4.4E-02	1.8E-01	1.7E-03	6.2E-04	2.0E-05	3.0E-05	1.3E-04

1558836_at	PLGLB1	5.0E-01	4.9E-01	5.0E-01	5.0E-01	6.0E-05	1.7E-02	2.0E-05	2.0E-05	1.0E-04
204285_s_at	PMAIP1	9.9E-01	5.0E-01	5.0E-01	3.0E-05	2.0E-05	2.0E-05	2.0E-05	2.0E-05	2.0E-05
204286_s_at	PMAIP1	9.2E-01	1.0E+00	5.0E-01	3.5E-04	4.0E-05	3.0E-05	3.0E-05	3.5E-05	2.0E-05
1554609_at	POLD3	5.0E-01	9.2E-01	1.0E+00	1.0E+00	1.0E+00	1.0E+00	1.0E+00	1.0E+00	1.0E+00
212226_s_at	PPAP2B	5.0E-01	5.0E-01	9.3E-01	5.0E-01	7.8E-05	2.0E-05	2.0E-05	2.3E-05	2.0E-05
212230_at	PPAP2B	5.0E-01	9.6E-01	6.7E-01	5.0E-01	4.9E-04	1.7E-04	2.0E-05	2.0E-05	2.0E-05
218273_s_at	PPM2C	5.0E-01	5.0E-01	5.0E-01	1.1E-01	1.7E-04	3.0E-05	3.0E-05	2.7E-05	2.0E-05
222572_at	PPM2C	5.0E-01	5.0E-01	5.0E-01	2.0E-02	2.0E-05	3.0E-05	2.0E-05	2.0E-05	2.0E-05
202014_at	PPP1R15A	5.0E-01	5.0E-01	2.4E-01	1.7E-03	1.7E-04	2.0E-05	2.0E-05	5.2E-05	2.7E-05
37028_at	PPP1R15A	2.8E-01	7.8E-02	5.0E-01	4.0E-03	1.0E-06	0.0E+00	0.0E+00	0.0E+00	0.0E+00
224692_at	PPP1R15B	5.0E-01	5.0E-01	4.8E-01	2.0E-05	2.0E-05	2.0E-05	2.0E-05	2.0E-05	2.0E-05
1552670_a_at	PPP1R3B	3.4E-01	5.0E-01	5.0E-01	3.0E-01	5.0E-01	2.1E-02	2.0E-05	4.0E-05	3.9E-04
225066_at	PPP2R2D	6.7E-01	1.5E-01	5.5E-04	1.7E-01	3.0E-03	3.2E-01	2.0E-05	2.0E-05	7.8E-05
201594_s_at	PPP4R1	5.0E-01	8.5E-01	4.8E-01	1.7E-02	1.0E-04	2.3E-05	2.0E-05	2.0E-05	2.0E-05
227510_x_at	PRO1073	1.0E+00	8.8E-05	2.2E-01	5.0E-01	7.8E-05	2.7E-05	2.0E-05	2.3E-05	2.0E-05
209586_s_at	PRUNE	5.0E-01	5.0E-01	5.0E-01	5.0E-01	1.0E+00	1.0E+00	1.0E+00	1.0E+00	1.0E+00
210988_s_at	PRUNE	5.0E-01	7.2E-01	5.0E-01	5.0E-01	1.0E+00	1.0E+00	1.0E+00	1.0E+00	1.0E+00
204748_at	PTGS2	2.0E-03	1.2E-03	4.6E-05	2.0E-05	2.0E-05	2.0E-05	2.0E-05	2.0E-05	2.0E-05
1554997_a_at	PTGS2	4.1E-02	2.5E-03	1.7E-04	2.0E-05	2.0E-05	2.0E-05	2.0E-05	2.0E-05	2.0E-05
208965_s_at	PYHIN1	2.7E-01	5.0E-01	5.0E-01	4.5E-01	3.9E-04	1.5E-04	2.0E-05	1.0E-04	4.0E-05
1557432_at	RASAL2	7.7E-01	5.8E-01	5.0E-01	8.0E-01	7.8E-03	5.0E-01	2.0E-05	6.2E-04	2.4E-04
223467_at	RASD1	5.8E-01	1.0E+00	1.3E-01	8.8E-05	1.1E-02	2.0E-05	2.0E-05	3.9E-04	1.0E-04
1568768_s_at	RBKS	9.0E-01	5.0E-01	1.0E-04	2.0E-05	2.0E-05	2.0E-05	2.0E-05	2.0E-05	2.0E-05
228455_at	RBM15	1.0E+00	5.0E-01	5.0E-01	1.0E+00	1.0E+00	1.0E+00	1.0E+00	1.0E+00	1.0E+00
202388_at	RGS2	1.0E+00	1.0E+00	5.0E-01	1.0E+00	2.0E-05	2.0E-05	2.0E-05	2.0E-05	2.0E-05
212099_at	RHOB	7.7E-01	1.0E+00	5.0E-01	2.0E-05	2.0E-05	2.0E-05	2.0E-05	2.0E-05	2.0E-05
1553962_s_at	RHOB	8.2E-01	1.0E+00	3.4E-01	2.0E-05	2.0E-05	2.0E-05	2.0E-05	2.0E-05	2.0E-05
223168_at	RHOU	5.0E-01	5.0E-01	1.0E+00	1.0E+00	1.0E+00	1.0E+00	1.0E+00	1.0E+00	1.0E+00
229285_at	RNASEL	3.4E-01	5.0E-01	8.3E-01	1.0E+00	1.0E+00	1.0E+00	1.0E+00	1.0E+00	1.0E+00
212724_at	RND3	1.0E+00	1.0E+00	5.0E-01	5.0E-01	2.5E-03	2.0E-05	2.0E-05	2.0E-05	2.0E-05
236114_at	RUNX1	7.3E-01	4.5E-01	5.0E-01	5.0E-01	2.0E-05	2.7E-04	7.8E-05	2.7E-05	7.7E-04
238909_at	S100A10	1.0E+00	9.8E-01	5.0E-01	8.4E-02	2.0E-05	3.0E-05	2.0E-05	2.0E-05	2.0E-05
204268_at	S100A2	5.0E-01	5.0E-01	1.9E-01	1.2E-01	7.8E-05	2.0E-05	2.0E-05	2.0E-05	2.0E-05
228923_at	S100A6	4.0E-01	4.3E-01	3.5E-01	3.0E-03	3.0E-05	2.3E-05	1.1E-04	1.0E-04	7.8E-05
203455_s_at	SAT1	5.0E-01	3.5E-04	2.0E-05	2.0E-05	2.0E-05	2.0E-05	2.0E-05	2.0E-05	2.0E-05
210592_s_at	SAT1	2.5E-02	2.0E-05	2.3E-05	2.0E-05	2.0E-05	2.0E-05	2.0E-05	2.0E-05	2.0E-05
213988_s_at	SAT1	3.6E-02	1.3E-03	4.6E-05	2.7E-05	2.3E-05	2.7E-05	2.0E-05	2.0E-05	2.7E-05
230333_at	SAT1	2.0E-05	2.0E-05	2.0E-05	2.0E-05	2.0E-05	2.0E-05	2.0E-05	2.0E-05	2.0E-05
235147_at	SATB2	9.8E-01	5.0E-01	1.0E+00	1.0E+00	9.9E-01	1.0E+00	1.0E+00	1.0E+00	1.0E+00
202071_at	SDC4	5.0E-01	4.8E-01	5.0E-01	2.3E-03	1.7E-04	3.0E-05	2.3E-05	2.0E-05	2.0E-05
234725_s_at	SEMA4B	3.7E-01	5.0E-01	5.0E-01	5.0E-01	3.9E-04	7.7E-04	8.8E-05	2.0E-05	4.0E-05
228398_at	SENP8	5.0E-01	8.1E-01	6.9E-01	9.9E-01	1.0E+00	1.0E+00	1.0E+00	1.0E+00	1.0E+00
1552684_a_at	SENP8	5.0E-01	5.0E-01	6.6E-01	9.3E-01	1.0E+00	1.0E+00	1.0E+00	1.0E+00	1.0E+00
1558937_s_at	SERF1A	1.0E+00	5.0E-01	8.1E-01	1.0E+00	1.0E+00	1.0E+00	1.0E+00	1.0E+00	1.0E+00
239213_at	SERPINB1	3.9E-01	5.0E-01	5.0E-01	6.3E-01	5.9E-03	4.1E-02	4.4E-04	2.7E-04	4.6E-05
209719_x_at	SERPINB3	1.9E-04	5.1E-02	1.8E-01	3.3E-02	2.0E-05	2.0E-05	2.0E-05	2.0E-05	2.0E-05
209720_s_at	SERPINB3	6.5E-01	4.1E-02	4.1E-02	6.8E-05	2.0E-05	2.0E-05	2.0E-05	2.0E-05	2.0E-05
210413_x_at	SERPINB4	1.8E-01	4.7E-01	2.3E-01	2.0E-02	4.6E-05	2.3E-05	2.0E-05	3.0E-05	2.0E-05
211906_s_at	SERPINB4	3.4E-01	7.4E-02	6.3E-01	5.0E-01	2.0E-05	2.0E-05	2.0E-05	2.0E-05	2.0E-05
223394_at	SERTAD1	2.1E-01	5.0E-01	1.2E-03	2.7E-04	2.0E-05	2.0E-05	2.0E-05	2.0E-05	2.0E-05
202656_s_at	SERTAD2	5.0E-01	5.0E-01	5.0E-01	5.0E-01	2.0E-05	2.7E-05	2.0E-05	2.0E-05	2.0E-05
202657_s_at	SERTAD2	5.0E-01	5.0E-01	5.0E-01	3.9E-04	2.0E-05	2.0E-05	2.0E-05	2.0E-05	2.0E-05
223196_s_at	SESN2	6.1E-01	5.0E-01	5.0E-01	5.0E-01	8.5E-01	8.2E-01	7.8E-05	1.7E-04	1.2E-03
209260_at	SFN	5.0E-01	5.0E-01	5.0E-01	1.5E-04	2.0E-05	2.0E-05	2.0E-05	2.0E-05	2.0E-05
33323_r_at	SFN	5.0E-01	5.0E-01	5.0E-01	5.0E-01	0.0E+00	0.0E+00	0.0E+00	0.0E+00	0.0E+00
201739_at	SGK	9.9E-01	1.0E+00	5.0E-01	2.7E-05	2.0E-05	2.0E-05	2.0E-05	2.0E-05	2.0E-05
242963_at	SGMS2	5.0E-01	5.0E-01	5.0E-01	2.5E-03	5.2E-05	2.0E-05	2.0E-05	2.3E-05	2.0E-05
1569263_at	SLC16A3	7.0E-02	2.6E-01	6.1E-01	5.0E-01	2.1E-04	6.2E-04	2.0E-05	3.5E-05	6.2E-04
230494_at	SLC20A1	1.0E+00	5.0E-01	7.4E-02	3.6E-02	2.0E-05	2.0E-05	2.0E-05	2.0E-05	2.0E-05
239474_at	SLC6A6	5.0E-01	2.8E-03	2.7E-02	2.9E-02	6.0E-05	7.4E-02	3.5E-05	2.7E-05	2.0E-03
223666_at	SNX5	5.0E-01	5.0E-01	3.3E-01	1.2E-03	7.8E-05	2.0E-05	1.5E-04	2.3E-05	5.2E-05
203372_s_at	SOCS2	7.0E-01	9.6E-01	5.0E-01	9.7E-01	8.5E-03	4.5E-03	2.0E-05	2.0E-05	6.8E-05
203373_at	SOCS2	7.3E-01	1.0E+00	5.0E-01	5.8E-01	2.8E-01	4.0E-05	2.0E-05	2.7E-05	2.0E-05
206359_at	SOCS3	5.0E-01	9.4E-01	5.0E-01	1.1E-03	6.0E-05	1.9E-04	8.8E-05	1.3E-04	7.8E-05
206360_s_at	SOCS3	5.0E-01	5.0E-01	5.0E-01	2.0E-03	1.5E-02	1.1E-02	1.5E-04	1.3E-04	2.7E-04
227697_at	SOCS3	9.7E-01	1.0E+00	5.0E-01	2.0E-05	2.0E-05	2.0E-05	2.0E-05	2.0E-05	2.0E-05
215078_at	SOD2	1.8E-01	2.0E-01	4.5E-01	6.2E-04	2.0E-05	2.0E-05	2.0E-05	5.2E-05	2.0E-05

215223_s_at	SOD2	2.3E-05	2.0E-05	2.0E-05	2.0E-05	2.0E-05	2.0E-05	2.0E-05	2.0E-05	2.0E-05
216841_s_at	SOD2	6.2E-02	3.5E-05	8.8E-05	2.0E-05	2.3E-05	2.0E-05	3.0E-05	2.0E-05	4.0E-05
202935_s_at	SOX9	5.0E-01	5.2E-01	5.0E-01	5.0E-01	1.0E-04	1.0E-04	2.3E-05	3.0E-05	6.0E-05
235680_at	STAT3	9.6E-01	7.6E-01	5.0E-01	8.9E-02	1.1E-04	5.0E-01	5.2E-05	1.7E-03	8.7E-04
243213_at	STAT3	5.0E-01	8.9E-02	5.0E-01	5.0E-01	1.2E-02	4.1E-02	2.0E-05	3.5E-04	2.7E-04
203439_s_at	STC2	8.6E-01	9.9E-01	5.0E-01	8.7E-01	5.1E-02	2.7E-02	2.0E-05	3.1E-04	6.2E-04
209238_at	STX3	5.0E-01	5.0E-01	5.0E-01	2.7E-05	2.0E-05	2.0E-05	2.0E-05	2.0E-05	2.0E-05
204067_at	SUOX	5.0E-01	5.0E-01	5.0E-01	9.9E-01	1.0E+00	1.0E+00	1.0E+00	1.0E+00	1.0E+00
230052_s_at	TA-NFKBH	5.0E-01	5.0E-01	1.9E-01	1.3E-02	4.9E-04	4.1E-03	4.6E-05	6.2E-04	7.7E-04
240206_at	TARS	5.0E-01	5.7E-01	5.0E-01	5.0E-01	1.0E-01	1.8E-03	6.8E-05	2.7E-04	3.0E-05
226625_at	TGFBR3	3.2E-01	5.0E-01	6.1E-01	1.0E+00	1.0E+00	1.0E+00	1.0E+00	1.0E+00	1.0E+00
230380_at	THAP2	6.3E-01	5.0E-01	3.0E-03	2.0E-05	2.0E-05	2.0E-05	2.0E-05	2.0E-05	2.0E-05
203887_s_at	THBD	5.0E-01	5.0E-01	1.6E-01	5.0E-01	2.2E-01	2.8E-03	4.9E-04	3.5E-05	1.9E-04
201107_s_at	THBS1	3.2E-01	9.8E-01	1.9E-01	3.3E-01	7.9E-02	5.4E-02	6.2E-04	4.9E-04	1.8E-03
201109_s_at	THBS1	5.0E-01	5.0E-01	2.9E-02	3.1E-02	1.5E-03	2.3E-05	4.6E-05	2.7E-05	1.3E-03
201110_s_at	THBS1	6.8E-01	5.0E-01	2.8E-03	3.8E-02	2.0E-05	2.0E-05	2.0E-05	3.0E-05	2.0E-05
213352_at	TMCC1	5.0E-01	8.2E-01	5.0E-01	1.0E+00	1.0E+00	1.0E+00	1.0E+00	1.0E+00	1.0E+00
227112_at	TMCC1	6.3E-01	1.0E+00	9.3E-01	1.0E+00	1.0E+00	1.0E+00	1.0E+00	1.0E+00	1.0E+00
213338_at	TMEM158	6.8E-01	9.4E-01	5.0E-01	5.0E-01	3.9E-01	4.6E-05	2.0E-05	6.0E-05	3.1E-04
218113_at	TMEM2	5.0E-01	5.0E-01	1.8E-02	1.1E-04	3.0E-05	2.3E-05	5.2E-05	3.5E-05	2.3E-05
224917_at	TMEM49	1.0E+00	5.0E-01	2.3E-05	2.0E-05	2.0E-05	2.0E-05	2.0E-05	2.0E-05	2.0E-05
231697_s_at	TMEM49	5.3E-01	5.0E-01	5.4E-02	1.1E-03	3.5E-05	2.0E-05	5.2E-05	6.8E-05	2.7E-05
1569003_at	TMEM49	6.7E-01	5.0E-01	2.0E-05	1.0E-04	2.0E-05	2.0E-05	2.0E-05	2.0E-05	2.0E-05
222449_at	TMEPAI	5.0E-01	6.0E-01	5.9E-03	1.7E-04	2.0E-05	2.4E-04	2.0E-05	2.0E-05	8.8E-05
202643_s_at	TNFAIP3	5.0E-01	5.0E-01	5.0E-01	5.0E-01	3.8E-02	2.0E-05	2.0E-05	2.0E-05	2.0E-05
202644_s_at	TNFAIP3	5.0E-01	6.5E-01	5.0E-01	5.0E-01	4.1E-03	2.0E-05	2.0E-05	2.0E-05	2.0E-05
227345_at	TNFRSF10D	5.6E-01	5.0E-01	4.0E-01	2.7E-01	4.9E-04	1.3E-04	2.0E-05	2.0E-05	4.6E-05
218368_s_at	TNFRSF12A	9.9E-01	1.0E+00	1.0E-04	2.3E-05	2.0E-05	2.0E-05	3.0E-05	2.3E-05	2.7E-05
202241_at	TRIB1	7.3E-01	9.8E-01	1.9E-01	2.0E-05	2.0E-05	2.0E-05	2.0E-05	2.0E-05	2.0E-05
1554250_s_at	TRIM73	7.8E-01	5.0E-01	7.4E-02	2.1E-01	1.1E-02	2.2E-01	2.0E-05	7.8E-05	6.8E-05
233771_at	TRIO	5.2E-01	5.0E-01	2.2E-01	4.1E-02	1.0E-02	3.0E-05	1.5E-04	4.6E-05	6.9E-04
204094_s_at	TSC22D2	5.0E-01	5.0E-01	5.0E-01	7.8E-05	2.0E-05	2.0E-05	2.0E-05	2.0E-05	2.0E-05
223282_at	TSHZ1	3.0E-01	2.4E-01	5.0E-01	8.4E-02	1.0E+00	1.0E+00	1.0E+00	1.0E+00	1.0E+00
1554588_a_at	TTC30B	7.3E-01	5.0E-01	5.0E-01	1.0E+00	1.0E+00	1.0E+00	1.0E+00	1.0E+00	1.0E+00
204141_at	TUBB2A	5.0E-01	8.8E-01	5.0E-01	5.0E-01	4.9E-04	4.9E-04	2.3E-05	6.0E-05	2.0E-05
201008_s_at	TXNIP	5.0E-01	5.0E-01	5.0E-01	1.0E+00	1.0E+00	1.0E+00	1.0E+00	1.0E+00	1.0E+00
201009_s_at	TXNIP	5.0E-01	5.0E-01	5.0E-01	1.0E+00	1.0E+00	1.0E+00	1.0E+00	1.0E+00	1.0E+00
201010_s_at	TXNIP	5.0E-01	5.0E-01	5.0E-01	9.9E-01	1.0E+00	1.0E+00	1.0E+00	1.0E+00	1.0E+00
204881_s_at	UGCG	5.0E-01	1.2E-01	5.8E-02	2.0E-05	2.3E-05	2.0E-05	2.0E-05	2.0E-05	2.0E-05
221765_at	UGCG	3.8E-01	9.1E-01	5.0E-01	5.1E-02	1.3E-04	5.2E-05	4.6E-05	2.7E-04	2.0E-05
224967_at	UGCG	5.0E-01	5.0E-01	5.0E-01	2.3E-05	2.0E-05	2.0E-05	2.0E-05	2.0E-05	2.0E-05
238542_at	ULBP2	7.4E-01	8.3E-01	5.0E-01	5.6E-01	5.0E-01	1.7E-01	1.9E-04	2.3E-05	2.0E-05
220370_s_at	USP36	5.0E-01	9.3E-01	3.4E-03	2.0E-05	2.0E-05	2.0E-05	2.0E-05	2.0E-05	2.0E-05
221704_s_at	VPS37B	3.9E-01	5.0E-01	4.1E-02	9.7E-04	3.0E-05	1.7E-03	1.5E-04	8.8E-05	4.6E-05
213425_at	WNT5A	8.3E-01	5.0E-01	5.0E-01	5.0E-01	1.0E+00	1.0E+00	1.0E+00	1.0E+00	1.0E+00
227501_at	WSB1	6.7E-01	5.0E-01	5.0E-01	5.0E-01	6.2E-04	1.9E-01	2.0E-05	4.6E-05	2.3E-03
215150_at	YOD1	5.0E-01	5.0E-01	5.0E-01	2.9E-02	1.3E-04	1.7E-04	7.8E-05	2.7E-05	2.3E-05
227309_at	YOD1	5.0E-01	5.2E-01	5.0E-01	4.0E-05	4.0E-05	2.0E-05	2.0E-05	2.0E-05	2.0E-05
227978_s_at	ZADH2	5.0E-01	5.0E-01	5.0E-01	8.1E-01	1.0E+00	1.0E+00	1.0E+00	1.0E+00	1.0E+00
218810_at	ZC3H12A	1.8E-02	5.0E-01	1.2E-03	3.1E-04	3.4E-03	7.8E-05	2.3E-05	2.1E-04	6.8E-05
231899_at	ZC3H12C	5.0E-01	5.0E-01	3.7E-01	2.3E-05	2.0E-05	2.0E-05	2.0E-05	2.0E-05	2.0E-05
1552283_s_at	ZDHHC11	5.0E-01	5.0E-01	3.4E-01	5.0E-01	6.2E-04	1.0E-02	5.2E-05	2.0E-05	6.0E-05
201531_at	ZFP36	4.2E-01	3.9E-01	1.3E-02	1.7E-04	4.0E-05	6.0E-05	4.6E-05	6.8E-05	4.0E-05
214670_at	ZKSCAN1	5.0E-01	9.1E-01	9.8E-01	1.0E+00	1.0E+00	1.0E+00	1.0E+00	1.0E+00	1.0E+00
1557953_at	ZKSCAN1	5.0E-01	5.0E-01	5.0E-01	1.0E+00	1.0E+00	1.0E+00	1.0E+00	1.0E+00	1.0E+00
222619_at	ZNF281	5.0E-01	5.0E-01	5.0E-01	2.4E-01	2.0E-05	2.0E-05	2.0E-05	2.0E-05	2.0E-05
211064_at	ZNF493	6.8E-01	5.0E-01	1.0E-01	8.1E-01	9.8E-01	1.0E+00	1.0E+00	1.0E+00	1.0E+00
227195_at	ZNF503	5.0E-01	5.0E-01	4.9E-03	6.5E-03	8.8E-05	4.0E-05	3.0E-05	2.3E-05	2.0E-05
235304_at	ZNF573	9.9E-01	6.7E-01	5.0E-01	1.0E+00	1.0E+00	1.0E+00	1.0E+00	1.0E+00	1.0E+00
228005_at	ZXDB	5.0E-01	1.0E+00	5.8E-01	5.0E-01	1.0E+00	1.0E+00	1.0E+00	1.0E+00	1.0E+00
