# Supplementary material for: Salmonella Typhimurium Type III Secretion Effectors Stimulate Innate Immune Responses in Cultured Epithelial Cells
Source: PLoS Pathog. 2009 Aug 7;5(8):e1000538. doi: 10.1371/journal.ppat.1000538 (PMC2714975; doi:10.1371/journal.ppat.1000538)
Supplement: Table S3 — List of genes stimulated by S. typhimurium (2 fold or more) that were also stimulated by other pathogens or agonists of the innate immune system. (0.05 MB PDF) [file ppat.1000538.s008.pdf]

Table S3: List of genes stimulated by S. typhimurium (2 fold or more) that were also stimulated by other pathogens or agonists of the innate immune system											
	Shigella flexneri(11,12)	Helicobacter pylori(1)	Yersinia enterocolitica(2)	Bordatella pertussis (4)	Pseudomonas aeruginosa (3,8)	Staphlococcus aureus(9)	Staphylococcus aureus(6)	Toxoplasma gondii(7)	Legionella pneumophila(10)	Different bacteria or bacterial components(5)	Core(15)
Gene Symbol	Caco-2	Gastric epithelial cells	HeLa	Lung Epithelial cells	lung carcinoma cell line	HUVEC	Human airway epithelial cells	Human foreskin broblasts	Macrophages	Macrophages	Many
CCL2 (MCP-1)			X	X	X	X		X		X	
CXCL2 (MIP2a, GRO2)	X		X	X	X	X	X	X	X	X	X
GEM			X		X			X		X	X
FOS	X	X			X		X		X		
JUN	X		X		X		X	X			X
EGR1			X				X	X			
ZFP36	X				X				X	X	X
SOD2			X			X		X	X	X	X
AREG /// LOC653193	X				X						
IL8	X		X	X	X	X	X		X	X	X
DUSP6	X	X						X	X		X
CXCL3 (MIP2b, GRO3)	X		X	X	X	X	X		X	X	X
CEBPD			X								
ID1	X										
KLF6 (COPEB)	X	X	X		X		X				X
ADM					X					X	X
AXUD1									X		
PTGS2	X		X		X		X	X		X	X
SERPINB1											X
ATF3	X	X	X		X			X			X
EREG	X							X			
FGB					X						
FOSL1		X	X								
IL11			X		X						X
DUSP5	X		X					X		X	X
SAT											X
JUNB	X		X		X	X	X	X		X	X
MAFB									X		
MAFF			X						X		
CYR61	X		X								
GADD45B									X		
CTGF	X		X								
IL1A			X				X		X		X
ISG20					X					X	X
PHLDA1			X		X			X	X		
EPHA2	X		X								
EGR3									X		
F2RL1	X										
PPP1R15A	X		X		X				X		
MAP3K8					X						X
TRIB1											X
AIM1											X
DUSP1	X				X				X	X	X
HES1	X										
IER3	X		X		X			X	X	X	X
CDKN1A			X						X		
GDF15		X									
MCL1	X		X								X
NFIL3											X
NR4A2			X		X		X	X			
STAT3							X				
TRIO			X								
NR4A1	X				X			X			
RGS2									X		
SERTAD1		X									
NR4A3	X				X						X
TNFAIP3	X		X	X	X	X	X	X	X	X	X
DKK1			X								
PLAUR					X					X	X
PBEF1					X			X	X	X	X
BHLHB2					X		X				
F3											X
MAFK	X										
BCL10									X		
DUSP8					X						X
DUSP4									X		X
PMAIP1			X		X			X			X
SLC6A6			X								
BCL3			X					X	X		X
C8orf4			X								
BIRC3			X		X		X		X	X	X
SLC16A3									X		
CASP4											X
HRH1			X								
SOCS2							X				
ADRB2											X
IFI16											X
ITGA2								X			
NFKBIA	X		X	X	X		X	X	X	X	X
MGC4677		X									
CREM											X
ETS2										X	X
THBS1	X		X								
EMP1			X								X
GFPT2			X								
KLF2			X				X				
LDLR	X									X	X
RHOB			X		X						X
FOSL2											X
IRF1	X		X		X	X		X		X	X
HSPB8		X									
LIF			X				X	X			X
UGCG					X						
PPAP2B					X						X
DUSP2									X	X	X
SDC4(Ryudocan core prot	X		X		X					X	X
RIS1			X								
SGK							X				
MT2A											X
STC2			X								
CEBPG							X				X
B4GALT5					X				X		
FGF2			X								
THBD								X	X		X
WNT5A											X

CCNG2	x										
FZD2			x								
Reference											
1	Genes Chromosomes Cancer. 2004 Aug;40(4):334-41.										
2	Cell Microbiol. 2004 Feb;6(2):129-41.										
3	Proc Natl Acad Sci U S A. 2000 Aug 15;97(17):9659-64.										
4	Proc Natl Acad Sci U S A. 2000 Dec 5;97(25):13847-52.										
5	Proc Natl Acad Sci U S A. 2002 Feb 5;99(3):1503-8.										
6	Physiol Genomics. 2005 Feb 10;20(3):244-55.										
7	J Biol Chem. 2001 Jun 29;276(26):24223-31										
8	Cell Microbiol. 2005 Nov;7(11):1635-46.										
9	Scand J Immunol. 2005 Jun;61(6):536-44.										
10	J Exp Med. 2006 Sep 4;203(9):2177-89										
11	J Biol Chem. 2003 Sep 5;278(36):33878-86										
12	Nature Immunology 8, 47 - 56 (2006)										
13	Nat Rev Microbiol. 2005 Apr;3(4):281-94. Review.										
